# Delineating the mechanisms of cerebellar degeneration in paediatric and adult primary mitochondrial disease

**DOI:** 10.1007/s00401-025-02891-6

**Published:** 2025-05-30

**Authors:** Laura A. Smith, Elizaveta A. Olkhova, Nichola Z. Lax, Yi Shiau Ng, Robert W. Taylor, Grainne S. Gorman, Daniel Erskine, Robert McFarland

**Affiliations:** 1 Mitochondrial Research Group, Faculty of Medical Sciences, Translational and Clinical Research Institute, Newcastle University, Newcastle Upon Tyne, NE2 4HH UK; 2 Translational Biosciences, Applied Sciences, Ellison Building, Northumbria University, Newcastle Upon Tyne, NE1 8ST UK; 3 NHS Highly Specialised Service for Rare Mitochondrial Disorders of Adults and Children, Newcastle Upon Tyne Hospitals NHS Foundation Trust, Newcastle Upon Tyne, NE1 4LP UK; 4 National Institute for Health and Care Research (NIHR), Newcastle Biomedical Research Centre (BRC), Newcastle University, Newcastle Upon Tyne, NE4 5PL UK

**Keywords:** mtDNA, DNA polymerase gamma (POLG), Alpers’ syndrome, MELAS, MERRF, Stroke-like episodes

## Abstract

Cerebellar ataxia is a frequent, debilitating neurological manifestation of primary mitochondrial disease and is associated with extensive neurodegeneration of the cerebellar cortical circuitry. However, the precise neuropathological mechanisms resulting in cerebellar degeneration in paediatric and adult forms of mitochondrial disease remain unclear. We therefore sought to perform a comparative neuropathological study using post-mortem cerebellar tissues from 28 paediatric and adult patients with pathogenic bi-allelic *POLG* variants and pathogenic mitochondrial DNA variants (m.3243A > G, m.8344A > G, m.13094T > C, and m.14709T > C), in addition to 18 neurologically normal control cases. We also sought to assess the prevalence and progression of cerebellar ataxia in an adult mitochondrial disease patient clinical cohort (*n* = 310) harbouring the same pathogenic variants as the post-mortem cases. Analysis of the clinical patient cohort revealed that at least 23.5–39.7% of adult patients with primary mitochondrial disease had predominantly cerebellar ataxia, with disease progression evident in 38.8% of patients. In the mitochondrial disease post-mortem tissue cohort, there was clear evidence of selective loss of inhibitory Purkinje cells, with corresponding oxidative phosphorylation protein deficiencies, which were more severe in comparison to mainly excitatory neuronal populations of the granule cell layer and dentate nucleus. Remaining Purkinje cells also demonstrated an increased expression of mitophagy-related proteins, including LC3B and BNIP3. Focal necrotic cerebellar cortical lesions, identified in eight patients, were characterised by decreased parvalbumin immunoreactivity, and sporadic c-Fos immunoreactivity was observed throughout the cerebellar cortices of 14 patients, suggestive of cerebellar cortical hyperactivity. Overall, these neuropathological features were more severe in the early onset POLG-related disease group and patients who had epilepsy. Our findings provide an important insight to the pathological mechanisms contributing to the degeneration of the cerebellar cortex in paediatric and adult forms of primary mitochondrial disease, highlighting an increased burden of pathology in early onset POLG-related disease which may have important prognostic and therapeutic implications.

**Supplementary Information:**

The online version contains supplementary material available at 10.1007/s00401-025-02891-6.

## Introduction

Primary mitochondrial diseases (PMD) comprise the largest group of inherited neurometabolic disorders and frequently present with progressive neurological impairments including ataxia, epilepsy and cognitive impairment [6]. Pathogenic variants in mitochondrial DNA (mtDNA) and nuclear DNA genes encoding proteins localised to the mitochondria [37], result in defective oxidative phosphorylation (OXPHOS) leading to impaired generation of ATP, disrupting cellular metabolism, neural function, and fundamental mitochondrial signalling pathways [5]. However, the precise pathological mechanisms causing the debilitating neurological phenotypes associated with paediatric and adult-onset PMD remain obscure, and consequently, specific therapies for patients are extremely limited.

Progressive ataxia is one of the most prevalent neurological manifestations in PMD, frequently affecting patients harbouring the m.3243A > G pathogenic variant in *MT-TL1* (NC_012920.1), which is the most common cause of mtDNA-encoded PMD and is associated with the maternally inherited syndrome termed mitochondrial encephalomyopathy, lactic acidosis and stroke-like episodes (MELAS) [29, 35]. Patients harbouring bi-allelic pathogenic variants in *POLG* (NM_002693.3), encoding the catalytic subunit of the mtDNA polymerase gamma (POLG)—the sole enzyme responsible for replication of mtDNA—also frequently develop mixed ataxia due to cerebellar atrophy and dorsal root ganglionopathy [28, 39]. The continuum of POLG-related diseases, which collectively represent the most prevalent group of nuclear-encoded PMDs, includes Alpers’ syndrome [1, 7, 28], which typically presents in infancy or adolescence with refractory epilepsy, and adult-onset POLG-related disease which may present with ataxia neuropathy spectrum (ANS), epilepsy, and chronic progressive external ophthalmoplegia (CPEO) [10, 11, 36]. However, cerebellar ataxia is also a prominent feature in patients harbouring pathogenic variants in other genes critical for mitochondrial function including *MT-TK, CABC1*, as well as large-scale mtDNA deletions and mtDNA rearrangements [23].

Neuropathological studies assessing post-mortem brain tissues from patients with PMD have demonstrated a particular vulnerability of the cerebellar cortex and dentate nucleus to neurodegeneration [4, 7, 8, 15, 19, 27, 47]. The depletion of inhibitory Purkinje cells, the sole neural output of the cerebellar cortex, in conjunction with OXPHOS protein deficiencies within remaining Purkinje cells, has been proposed to alter the inhibitory restraint within the cerebellar cortical circuitry and contribute to ataxic symptoms in patients with PMD [4, 8, 19]. However, there is a paucity of comparative studies evaluating the precise neuropathological features of cerebellar degeneration in early onset PMD in comparison to adults with PMD, and between different PMD genotypes.

Stroke-like episodes, defined as a subacute onset of brain impairment caused by seizure activity and associated with an evolving neurological phenotype determined by location of the initial lesion [30], have been reported in paediatric and adult patients with mtDNA and POLG-related diseases. Neuroimaging typically identifies confluent, cross-vascular territory changes in the posterior brain cortices in patients presenting with stroke-like episodes and focal seizures, and more recently, subacute magnetic resonance imaging (MRI) scan changes in the cerebellar cortex, referred as cross-cerebellar diaschisis, have been reported [10, 31]. These events are hypothesised to be driven by severe metabolic impairment and seizure-associated neuronal hyperexcitability due to a deficit in inhibitory neuronal function [14, 30]. A recent post-mortem neuropathological study demonstrated that in an adult PMD patient cohort, the cerebellum harboured the highest frequency of focal necrotic stroke-like lesions in comparison to other brain regions [31], suggesting that the cerebellar cortex may be particularly vulnerable to OXPHOS deficiency. However, the severity of the neuropathological features of stroke-like lesions between different paediatric and adult PMD remain poorly understood.

In this study, we sought to perform a comparative neuropathological study to characterise the specific features of cerebellar degeneration in a large post-mortem tissue cohort obtained from paediatric and adult patients with POLG-related and mtDNA PMD. We characterised the neuropathological features of focal necrotic lesions in the cerebellum and explored the differential cellular vulnerabilities to mitochondrial OXPHOS protein deficiencies and changes in the expression of mitophagy and autophagy proteins. In addition, we also quantified the prevalence and progression of cerebellar ataxia in an adult PMD cohort. Crucially, our findings provide an important insight to the pathomechanisms which may underpin cerebellar dysfunction in patients with PMD, including a notable susceptibility of Purkinje cells to mitochondrial impairments and degeneration. Importantly, our study highlights a conspicuous vulnerability of the cerebellar cortex in early onset POLG-related disease which we propose is associated with the severe clinical severity of this form of PMD.

## Materials and methods

### Mitochondrial disease patient clinical cohort

To quantify the prevalence and progression of cerebellar ataxia in adult patients with PMD, we accessed data from the Wellcome Centre for Mitochondrial Research Patient Cohort UK, recorded between 2005 and 2024. This included ataxia clinical rating scores, assessed by the Newcastle Mitochondrial Disease Adult Scale (NMDAS) [40], for 310 patients with PMD harbouring bi-allelic pathogenic variants in *POLG* and mtDNA variants (Supplementary Table 1). Patients who scored > 2 for Question 6 and < 2 for Question seven (Section III NMDAS) were considered to have predominantly cerebellar ataxia. NMDAS scores from two appointments were included for analyses for each patient, including the patient’s first and most recent assessments.

### Post-mortem tissue cohorts

Formalin-fixed paraffin-embedded (FFPE) post-mortem cerebellum tissues were obtained from the Newcastle Brain Tissue Resource, BRAIN UK Network, Edinburgh Brain and Tissue Bank, Medical University of Vienna, and the National Institutes of Health NeuroBioBank at the University of Maryland (Supplementary Tables 2 and 3). Ethical approval was granted by the brain banks.

Tissues were obtained from 10 paediatric and 18 adult patients with PMD (Table 1 and Supplementary Table 2). This included six paediatric patients with clinically and neuropathologically defined Alpers’ syndrome, four paediatric patients with genetically confirmed Alpers’ syndrome, and three young adult patients with an Alpers-like POLG-related encephalopathy phenotype. These younger patients (age range: 14 months–28 years/y) were grouped together for analyses (referred to as patients with Alpers’ syndrome) and were compared to five older patients with bi-allelic POLG-related disease (age range: 46–79y), who had a longer disease duration or presented with symptoms later in life (termed late-POLG). Ten adult patients harbouring pathogenic variants in mtDNA (m.3243A > G, m.8344A > G, m.14709T > C, and m.13094T > C) were also included for comparison (age range: 20–64y). 12 of 28 PMD patient cerebellar tissues were included in previous neuropathological studies (Supplementary Table 2) [4, 8, 19, 20].

**Table 1:** Clinical details of PMD patient post-mortem cohort

Case	Clinical diagnosis	Molecular genetics	Sex	Age at onset	Age at death	Approx. disease duration	Ataxia	Epilepsy	Reported stroke-like episode	Neuroimaging
Pt.01^a^	Alpers’ syndrome	p.[Ala467Thr]/p.[Gly848Ser]	F	11 m	14 m	3 m		+		Mild generalised cerebral atrophy
Pt.02^a^	Alpers’ syndrome	Unknown	M	6 m	17 m	11 m		+		Gross cerebral atrophy
Pt.03^a^	Alpers’ syndrome	Unknown	F	12 m	18 m	6 m		+		Severe atrophy and extensive periventricular lucencies
Pt.04^a^	Alpers’ syndrome	Unknown	M	1.5 y	2.8 y	1.3 y		+		Severe atrophy
Pt.05	Alpers’ syndrome	p.[Cys418Arg]/p.[Ala467Thr]	M	2.5 y	3.2 y	8 m		+		Right-sided acute infarction in the frontal lobe and thalamus; mild supratentorial volume loss
Pt.06^a^	Alpers’ syndrome	Unknown	F	2 m	4 y	4.8 y		+		N/A
Pt.07^a^	Alpers’ syndrome	p.[Trp748Ser]/p.[Trp748Ser]	F	6 m	7 y	6.5 y	+	+		N/A
Pt.08	Alpers’ syndrome	p.[Ala467Thr]/p.[Gly848Ser]	M	2 y	11	9 y	+	+		N/A
Pt.09^a^	Alpers’ syndrome	Unknown	M	6 y	12	6 y	+	+		Progressive cerebral atrophy
Pt.10^a^	Alpers’ syndrome	Unknown	F	6 m	14 y	13 y	+	+		Marked symmetrical atrophy
Pt.11	Alpers’ syndrome	p.[Ala467Thr]/p.[Ala467Thr]	F	18 y	23 y	5 y	+	+	+	Bilateral occipital and right parietal stroke-like lesions. Right occipital, right temporal and right parietal atrophy
Pt.12	Alpers’ syndrome	p.[Ala467Thr]/p.[Trp748Ser]	F	20 y	24 y	4 y	+	+	+	Left occipital, parietal, thalamic, medulla and cerebellar signal abnormalities
Pt.13	Alpers’ syndrome	p.[Ala467Thr]/p.[Trp748Ser]	F	16 y	28 y	12 y	+	+	+	Bilateral frontal, parietal, occipital stroke-like lesions; cross-cerebellar diaschisis
Pt.14	Late-POLG	p.[Ala467Thr]/p.[Ala467Thr]	F	N/A	46 y	N/A	+		–	N/A
Pt.15	Late-POLG	p.[Ala467Thr]/p.[X1240Cys]	M	15 y	50 y	35 y	+	+		N/A
Pt.16	Late-POLG	p.[Trp748Ser]/p.[Arg1096Cys]	M	N/A	55 y	N/A	+	+	–	Generalised atrophy; subcortical and deep white matter T2 hyperintensities; encephalomalacia in the lateral right temporal lobe and right frontal cortex
Pt.17	Late-POLG	p.[Gly848Ser]/p.[Ser1104Cys]	M	22 y	59 y	37 y	+	–	–	Small foci signal abnormalities in cerebral white matter likely caused by chronic small vessel disease
Pt.18	Late-POLG	p.[Thr251Ile]/p.[Pro587Leu], p.[Ala467Thr]	M	N/A	79 y	N/A	+	–	–	Right occipital, parietal, temporal lobe changes
Pt.19	MELAS	m.3243A > G	F	10 y	20 y	10 y	+	+	+	N/A
Pt.20	MELAS	m.3243A > G	F	20 y	42 y	12 y	+		+	N/A
Pt.21	MELAS	m.3243A > G	M	37 y	45 y	8 y	+	+	+	N/A
Pt.22	MELAS	m.3243A > G	F	45 y	64 y	19 y	+	+	+	Right temporal and parietal lobes and left insular cortex stroke-like lesions
Pt.23	MERRF	m.8344A > G	M	14 y	31 y	17 y	+	+	–	N/A
Pt.24	MERRF	m.8344A > G	F	18 y	42 y	24 y	+	+		N/A
Pt.25	MERRF/MELAS	m.8344A > G	M	38 y	58 y	20 y		+	+	Involvement of both hemispheres and basal ganglia
Pt.26	MERRF	m.8344A > G	F	38 y	64 y	26 y	+	+	–	Moderate cerebellar atrophy and mild cerebral atrophy
Pt.27	MELAS/ Leigh Syndrome	m.13094T > C	F	32 y	34 y	2 y	–	+	+	Right subthalamus, upper midbrain and superior colliculus signal abnormality;
Pt.28	Cognitive impairment, peripheral neuropathy, diabetes mellitus, CPEO, IHD, PVD	m.14709T > C	M	34 y	55 y	21 y	–	–	–	N/A

*POLG* DNA polymerase gamma, *MELAS* Mitochondrial encephalomyopathy with lactic acidosis and stroke-like episodes, *CPEO* Chronic progressive external ophthalmoplegia, *IHD* ischaemic heart disease, *PVD* peripheral vascular disease, *F* Female, *M* Male, *y years*, *m* months, *N/A* not available, RefSeq NM_002693.3 and NC_012920.1

^a^Denotes historical patients with limited availability of clinical and neuroimaging data

The Alpers’ syndrome patient group (Pt.01–Pt.13, median age: 7 years) was compared to nine neurologically normal controls (Ct.01–Ct.09, median age: 14.5 years) matched for age at death (*P* = 0.883, Mann–Whitney), sex distribution (*P* = 0.999, Fisher’s exact test), post-mortem interval (*P* = 0.371, *t*-test) and formalin fixation length (*P* = 0.547, Mann–Whitney) (Supplementary Table 3). Fifteen adult patients with PMD (Pt.14–Pt.28, median age: 50 years) were matched for age (*P* = 0.0932, *t*-test), sex distribution (*P* = 0.389, Fisher’s exact test), post-mortem interval (*P* = 0.693, Mann–Whitney), and tissue fixation length (*P* = 0.512, Mann–Whitney) to eight neurologically normal controls (Ct.10–Ct.18, median age: 59 years).

### Quantification of Purkinje cell density and molecular cell layer width

Histological stains including Cresyl fast violet (CFV) and Luxol fast blue with haematoxylin and eosin (LFB-HE) were employed using 10 µm-thick FFPE sections to qualitatively assess neuropathological features and to identify Purkinje cells for neuronal density quantification in FFPE cerebellar tissues.

An Olympus BX51 stereology microscope, equipped with a motorised stage, 4–100 × Apo objectives, and a CCD colour camera, was employed to examine histological staining. As previously described [8], the density of Purkinje cells was quantified by manually outlining the Purkinje cell layer (residing between the molecular cell layer and granular cell layer) at × 4 magnification and counting cells using the Meander scan function at × 40 magnification in Stereo Investigator (MBF Biosciences software). Purkinje cells with a defined cell body and typical morphology (approximately 200–1000 µm^2^ round soma) residing within the linear layer were counted and the density was calculated (number of cells/mm^2^). The thickness of the molecular cell layer was also analysed by measuring the distance (µm) from the pia mater to the Purkinje cell layer. At least 20 measurements were recorded per case by randomly sampling multiple cerebellar folia, at the midpoint between the sulcus and crest of the folia.

### Immunohistochemical characterisation of focal necrotic lesions and autophagy-related proteins

To characterise and define focal necrotic cerebellar lesions, immunohistochemistry was performed to identify parvalbumin (calcium-binding protein expressed by Purkinje cells), GFAP (glial fibrillary acidic protein; reactive astrocytes and Bergmann glia), HLA-DP, DQ, DR (human leucocyte antigen; activated microglia), and c-Fos (marker of neuronal hyperactivity) using 5 µm-thick FFPE sections, as previously described (Supplementary Table 3 and Supplementary Information) [20, 43]. Focal necrotic lesions were identified based on defined demarcated regions of the cerebellar cortex, varying in size (approximately 10–60 mm^2^) and involving single or multiple cerebellar folia which were affected by severe neurodegeneration and cortical thinning, typically affecting the Purkinje cell layer (involving complete, or almost complete, Purkinje cell loss) and the molecular cell and granular cell layers (involving severe thinning, cell loss and gliosis) (Supplementary Fig. 1). Serial sections containing focal necrotic lesions were imaged using a motorised Zeiss Axioscan 7 slide scanner and ZEN imaging software, or were tiled using a Zeiss AxioImager and Zen Blue software, at × 10 magnification.

Since mitochondrial OXPHOS protein deficiencies and increased mitochondrial mass have previously been reported in tissues from patients with PMD [4, 8, 43], the spatial expression of autophagy-related proteins in cerebellar tissues was assessed by performing immunohistochemistry to identify BNIP3 (BCL1 interacting protein; mitophagy receptor), LC3B (microtubule-associated protein 1A/1B-light chain 3; autophagosome membrane protein), LAMP2 (lysosome-associated membrane protein 2), and p62 (sequestosome-1; autophagy adaptor protein). The expressions of these autophagy proteins and c-Fos were assessed using an Olympus BX51 stereology microscope. A qualitative scoring system was employed based on the abundance and intensity of immunoreactive cells in PMD patient tissues relative to age-matched control tissues with similar formalin fixation durations (Supplementary Fig. 2).

### Multiplex immunofluorescence to identify mitochondrial OXPHOS proteins

To quantify the abundance of mitochondrial OXPHOS proteins in cerebellar tissues, a previously optimised multiplex immunofluorescence assay was performed, which identifies the nuclear DNA-encoded complex I subunit NDUFB8 (NADH:ubiquinone oxidoreductase subunit B8), the mtDNA-encoded complex IV subunit COXI (cytochrome *c* oxidase subunit I), and porin (VDAC1; mitochondrial mass marker) (Supplementary Information) [8]. The expressions of NDUFB8 and COXI subunits are integral for the maintenance of function and structure of complexes I and IV, respectively [46, 50], and are markedly decreased in PMD patient cerebellar and cortical tissues [2, 4, 8, 18, 32, 43, 44]. Cerebellar sections were also incubated with an anti-parvalbumin antibody to identify Purkinje cells. Only tissues fixed in formalin for less than 1 year were included for the immunofluorescence experiments, since formalin fixation durations longer than this may affect the antigenicity of target proteins [49]. All primary and secondary antibodies used for this immunofluorescence assay are listed in Supplementary Table 4 and 5, respectively.

Immunofluorescent sections were imaged at × 20 magnification using an inverted ZEISS LSM800 confocal microscope and ZEISS Zen Blue software. Image capture settings for each laser (405 nm, 488 nm, 561 nm, and 640 nm) were optimised and maintained for all sections. Approximately 50 Purkinje cells were randomly imaged across non-lesioned regions of the superior and inferior cerebellar cortex, per case. Purkinje cells were identified based on morphology, localisation within the linear Purkinje cell layer, and positive parvalbumin and porin staining. The expression of porin was localised within the cytoplasm of cells and punctate labelling was observed with typical mitochondrial network morphology identified at high magnification. Adjacent regions of the granular cell layer and neurons of the dentate nucleus were also imaged based on porin staining. However, due to differences in tissue processing and dissection protocols between brain bank centres, the dentate tissues were only available for 15 patients and 12 control cases.

### Quadruple immunofluorescence analysis

Using ZEISS Zen Blue Desk software, individual Purkinje cells were manually outlined, and the intensity of each protein (NDUFB8, COXI, porin, and parvalbumin) and area (µm^2^) of each cell were measured [8]. As a comparison to inhibitory Purkinje cells, the intensity of the mitochondrial proteins was also measured within adjacent regions of the granular cell layer (which is mostly composed of excitatory neurons) and within neurons of the dentate nucleus.

To determine the abundance of complex I and complex IV subunits relative to mitochondrial mass, the intensities of NDUFB8, COXI, and porin were first log-transformed to normalise the data. The ratios of NDUFB8 to porin, and COXI to porin, were then calculated per cell. Using control NDUFB8/Porin and COXI/Porin ratios, the levels of OXPHOS protein expression in Purkinje cells were determined by calculating z-scores based on standard deviation limits, as previously described [38]. To infer changes to mitochondrial mass and parvalbumin protein expression, z-scores were also calculated using the intensity values of porin and parvalbumin, respectively.

### Statistical analyses

GraphPad Prism 9.0–10.0 (GraphPad Software, Inc., La Jolla, California) and R (R Core Team, 2020) were used for all analyses. Data were assessed for normality using the Shapiro–Wilk test and Q–Q plots were visually inspected. Demographic data were analysed using unpaired two-tailed Student’s *t* or Mann–Whitney *U* tests in GraphPad Prism. The Purkinje cell density data were analysed using a linear regression model in R. The molecular cell layer width data and all OXPHOS data were analysed using a linear mixed-effects model, accounting for the total number of measurements and total numbers of cells analysed per case, and per group, respectively, in R. Significance level was set to *P* < 0.05.

## Results

### Clinical details

In a cohort of 310 adult patients with PMD (*n* = 22 bi-allelic *POLG*, *n* = 248 m.3243A > G, *n* = 34 m.8344A > G, *n* = 6 m.14709T > C, Supplementary Table 1), 73 patients (23.5%) had predominantly cerebellar ataxia, assessed by the NMDAS,^23^ at the first appointment (Table 2). In comparison to the most recent follow-up assessment, cerebellar ataxia had progressed in 38.8% of patients, and a total of 123 of 310 patients (39.7%) presented with cerebellar ataxia. Mixed sensory and cerebellar ataxia was common in the POLG disease patient group, affecting 36.4% and 81.8% of patients at the first and most recent assessment, respectively.

**Table 2:** Prevalence of ataxia in adult patients with PMD

Genotype	Total number of patients	Patients with predominant cerebellar ataxia			Patients with mixed sensory and cerebellar ataxia		
		First assessment	Recent assessment	Progression	First assessment	Recent assessment	Progression
m.14709T > C	6	3/6 (50%)	3/6 (50%)	2/3 (66.6%)	0/6 (0%)	0/6 (0%)	NA
m.3243A > G	248	51/248 (20.6%)	100/248 (40.3%)	19/51 (37.3%)	0/34 (0%)	0/34 (0%)	NA
m.8344A > G	34	13/34 (38.2%)	19/34 (55.9%)	5/13 (38.5%)	0/34 (0%)	2/34 (5.9%)	NA
Bi-allelic *POLG*	22	6/22 (27.3%)	1/22 (4.5%)	NA	8/22(36.4%)	18/22 (81.8%)	6/8 (75%)
All genotypes	310	73/310 (23.5%)	123/310 (39.7%)	26/67 (38.8%)	8/310 (2.6%)	20/310 (6.5%)	6/8 (75%)

Clinical data were provided by the Wellcome Centre Mitochondrial Disease Patient Cohort UK for 310 adult patients. Ataxia and neuropathy were assessed using the Newcastle Mitochondrial Disease Adult Scale (NMDAS). Scores from Section III Question 6 (cerebellar ataxia) and Question 7 (neuropathy) were analysed. Predominant cerebellar ataxia: scores 2–5 for question 6 and 0–2 for question 7. Mixed sensory and cerebellar ataxia: scores 2–5 for question 6 and 3–5 for question 7. Median interval was 5 years, and mean interval was 6.2 years between the first and most recent follow-up assessment. All *POLG* patients with cerebellar ataxia at the first assessment had mixed sensory and cerebellar ataxia at the follow-up assessment; thus, progression of predominantly cerebellar ataxia could not be assessed (*NA* not applicable)

The PMD post-mortem tissue cohort consisted of 13 patients with Alpers’ syndrome (Patient 1 – 13), five adult patients with bi-allelic late-POLG disease (Patient 14–18), and ten adult patients with mtDNA disease (Patient 19–28: m.3243A > G, *n* = 4; m.8344A > G, *n* = 4; m.13094T > C, *n* = 1; m.14709T > C, *n* = 1) (Table 1). The mean age of disease onset was significantly younger (*P* < 0.0001), and the duration of disease was significantly shorter (*P* < 0.001), in the Alpers’ syndrome patient group (mean onset: 5.3 years, mean duration: 4.9 years) in comparison to the late-POLG and mtDNA disease group (mean onset: 26.9 years, mean duration: 19.3 years).

In this patient cohort, 19 of 28 patients displayed clinical signs of ataxia, including 7 patients with Alpers’ syndrome and 12 adult patients with POLG and mtDNA PMD. However, ataxia may not have been assessed in young infants who had not yet acquired key motor developmental milestones. Refractory epilepsy, which included frequent episodes of *status epilepticus*, was a defining feature of all patients with Alpers’ syndrome. Developmental delay and/or psychomotor regression, cortical blindness, and hepatic dysfunction were also common in these younger patients. In the adult PMD cohort, two of five patients with bi-allelic POLG-related disease and eight of ten patients with mtDNA disease had epilepsy. Stroke-like episodes were confirmed through the identification of cerebral stroke-like lesions in nine patients, and subacute signal abnormalities involving the cerebellar cortex were reported in two patients (Table 1).

### Cerebellar neuropathology

**Fig. 1:**
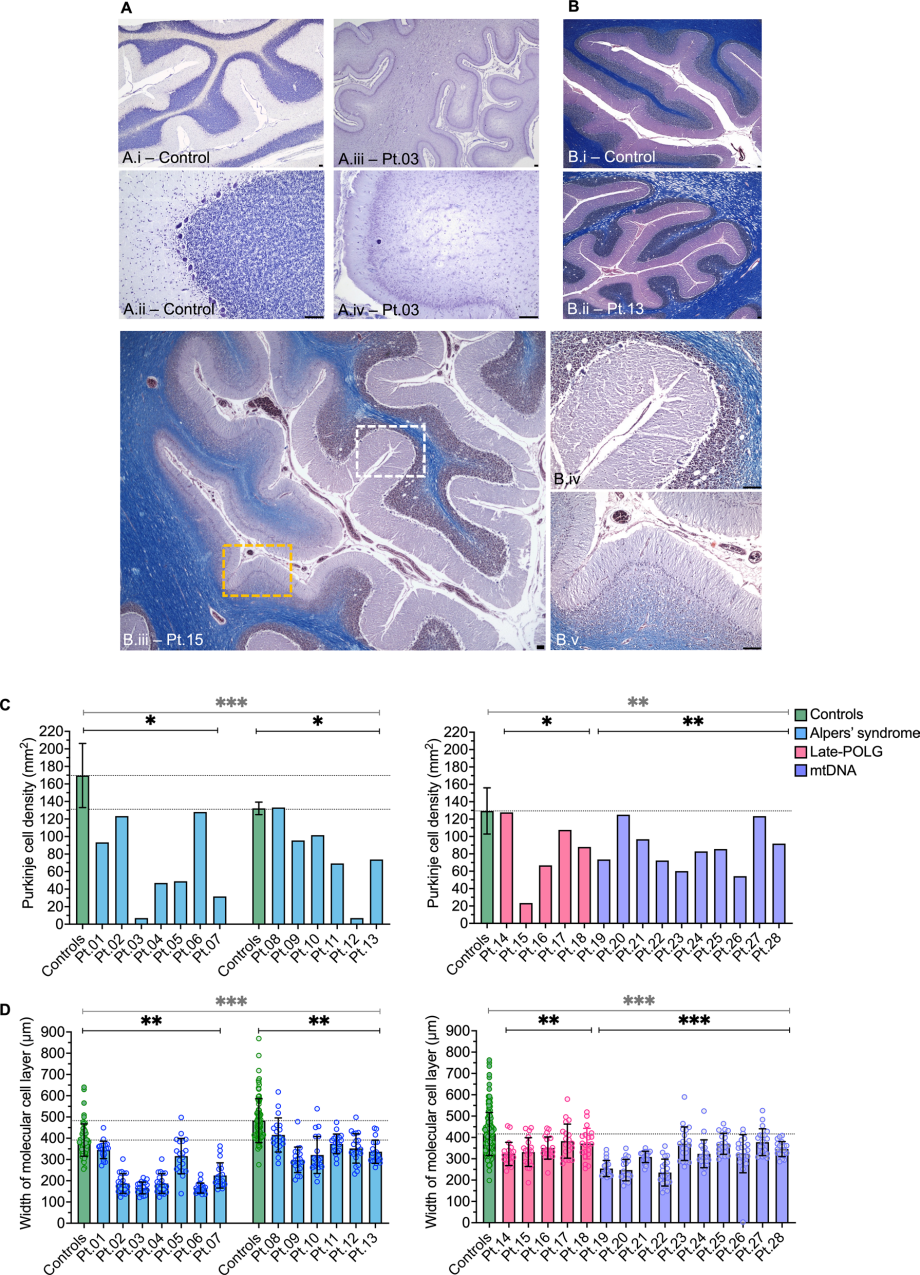
Degeneration of the cerebellar cortex in patients with mitochondrial disease. **A** Cresyl fast violet (CFV) stain reveals intact cerebellar cortex and preserved Purkinje cell density in control tissue (**i**–**ii**). However, cerebellar tissue from a patient with Alpers’ syndrome (Pt.03, **iii**–**iv**) demonstrates severe thinning of the molecular cell layer, loss of Purkinje cells and granular cell layer depletion. **B** Luxol fast blue, Haematoxylin and Eosin (LFB H&E) stain reveals intact myelin (blue) in control tissue (**i**). However, cerebellar tissue from a young adult patient with POLG-related disease (Pt.13, **ii**) demonstrates spongiosis in the subcortical white matter. (**iii**) A focal necrotic lesion characterised by total Purkinje cell loss and severe thinning of the cortical layers is presented from an adult patient with late-POLG disease (Pt.15). Preserved cortex (white box—**iv**) is outlined in the cerebellar folia adjacent to the lesioned cortex (yellow box—**v**). Scale bars = 100 μm. **C** Purkinje cell densities and **D** molecular cell layer width measurements (*n* = 20 per case) were analysed using a linear regression model (**C**) and linear mixed regression model (**D**) respectively. Grey bar and asterisk indicate comparisons where data from all patients with Alpers’ syndrome are pooled, and all late-onset POLG and mtDNA patients are pooled and compared to age-matched control groups. Black bar and asterisk indicate further comparisons splitting the Alpers’ syndrome patients into two groups based on age, and comparisons between the late-onset POLG group to controls, and mtDNA group to controls. *P < 0.05, **P < 0.01, ***P < 0.001

In line with previous neuropathological studies [8, 19], assessment of post-mortem cerebellar tissues revealed variable degrees of degeneration in the PMD tissue cohort involving a decreased abundance of Purkinje cells, thinning of the molecular cell layer, and depletion of the granular cell layer (Fig. 1A–B, Supplementary Table 6). Degeneration of Purkinje cells was typically accompanied by Bergmann gliosis and microgliosis predominantly affecting both the molecular cell layer and Purkinje cell layer. The white matter mostly appeared normal, except for regions affected by severe neurodegeneration where loss of myelin, evident by decreased Luxol fast blue staining, and vacuolation in the subcortical white matter were observed (Fig. 1B). Assessment of the deep cerebellar nuclei demonstrated an apparent moderate loss of neurons and gliosis in the dentate nucleus, although dentate tissue was only available for analysis in 15 cases.

Qualitative assessment of cerebellar tissues revealed visibly more severe atrophy of the cerebellar cortex in patients with Alpers’ syndrome relative to age-matched controls (Fig. 1A iii), in comparison to adult patients with POLG-related disease and mtDNA disease relative to adult controls (Fig. 1B ii, iii). Therefore, we sought to characterise and compare the severity of neuropathological features in cerebellar tissues across the PMD patient groups, with the aim of identifying shared and distinct pathomechanisms of cerebellar degeneration.

### Decreased Purkinje cell density and molecular cell layer width

Quantification of the density of Purkinje cells in control and PMD patient tissues revealed a significantly decreased abundance of Purkinje cells (*P* < 0.05) in the Alpers’ syndrome, late-POLG and mtDNA disease patient groups relative to age-matched controls (Fig. 1C). Interestingly, Purkinje cell loss tended to be more severe in PMD patients with epilepsy. We also quantified the surface area of individual Purkinje cells which revealed in cerebellar tissues from patients with Alpers’ syndrome and mtDNA disease, and Purkinje cells were significantly smaller compared to the size of control Purkinje cells suggestive of atrophy (*P* < 0.05) (Supplementary Fig. 3). Quantification of the width of the molecular cell layer revealed a significantly thinner layer in all PMD patient groups compared to controls (*P* < 0.01). However, the density and size of Purkinje cells and the width of the molecular cell layer did not differ significantly between different PMD patient groups (*P* > 0.05).

### Focal lesioned cerebellar cortex is characterised by decreased parvalbumin immunoreactivity

**Fig. 2:**
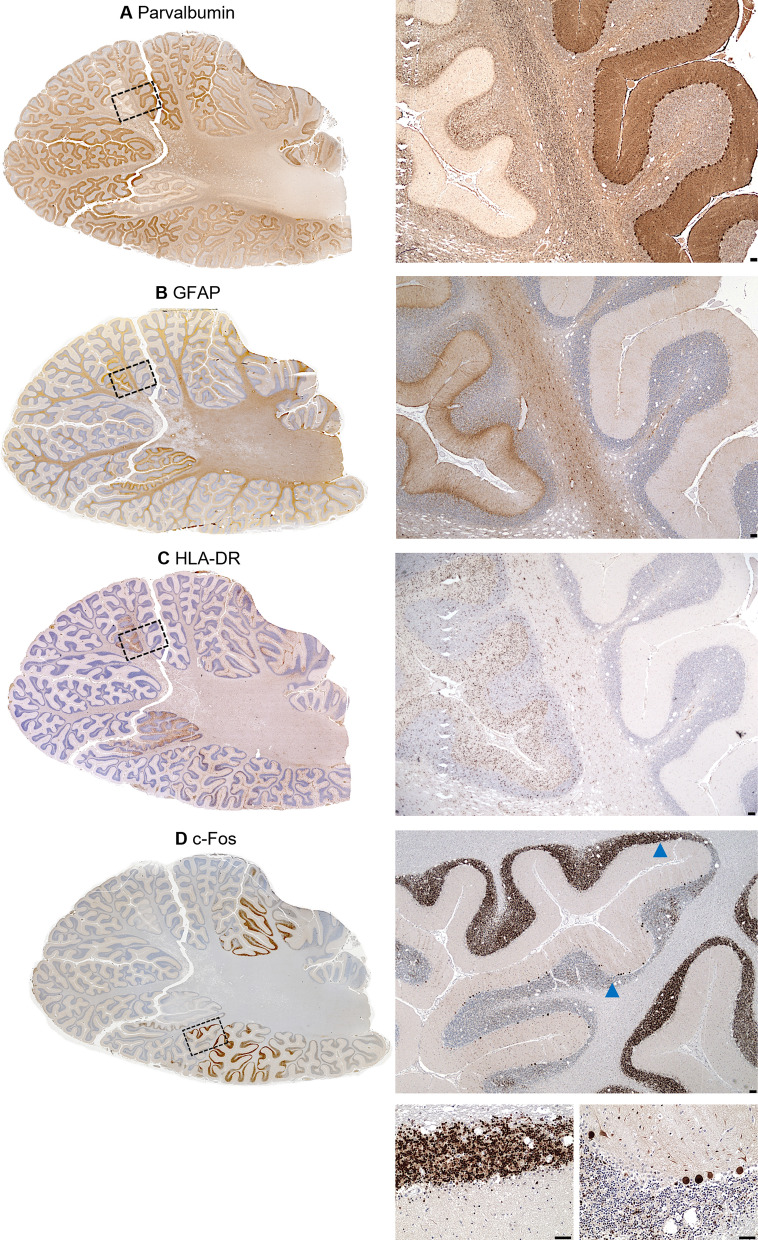
Pathological characterisation of focal lesions in the cerebellar cortex in POLG-related disease. Slide-scanned serial cerebellar cortical sections from a young adult patient (Pt.13) harbouring pathogenic variants in *POLG* (p.[Ala467Thr]/p.[Trp748Ser]) demonstrate focal lesions (example identified by dashed black box) characterised by: total Purkinje cell loss and decreased parvalbumin immunoreactivity (**a**), increased glial fibrillary acidic protein (GFAP; marker of reactive astrocytes) immunoreactivity (**b**), and increased HLA-DR (marker of activated microglia) immunoreactivity (**c**), in comparison to adjacent non-lesioned cortex. Increased c-Fos immunoreactivity (marker of neuronal hyperactivity) is observed in multiple cerebellar folia, labelling demarcated non-lesioned regions of the granular cell layer, molecular cell layer, and Purkinje cells (**d**). Blue arrow heads (**d**) indicate the region of cerebellar cortex which was imaged at a higher magnification. All sections were counterstained with Haematoxylin. Scale bars = 100 μm

Focal necrotic lesions in the cerebellum were identified in eight patients including four patients in the Alpers’ syndrome group, two adult patients with late-POLG disease, and two adult patients with mtDNA disease, all of whom had epilepsy (Table 3 and Supplementary Table 6). These lesions were characterised by demarcated regions of the cerebellar cortex affected by severe neurodegeneration, typically affecting all three cortical layers. In all cases, these lesions were identified by severely decreased parvalbumin-positive immunoreactivity in comparison to adjacent non-lesioned preserved cortex with intact parvalbumin staining (Fig. 2, Supplementary Figs. 4 and 5). This novel observation suggests that parvalbumin is a sensitive and reliable marker in the neuropathological identification of focal necrotic lesions in the cerebellar cortex of patients with PMD.

**Table 3:** Summary of cerebellar neuropathological features in PMD patient tissues

Patients		Focal necrotic lesion*	Purkinje cell density (% loss)	% NDUFB8 deficiency (z < -3)	% COXI deficiency (z < -3)	% Porin increase (z > 2)	LC3B	BNIP3	p62	c-Fos
Alpers’syndrome/Early-POLG disease	Pt.01		93.4 (45%)	71.4	54.4	85.6	+ + +	–	–	–
	Pt.02		123.4 (27.3%)	0.0	58.2	58.2	–	–	–	+
	Pt.03	Yes	7.0 (95.8%)	NA	NA	NA	+	NA	–	+
	Pt.04		47.1 (72.3%)	0.0	15.9	22.7	+ +	+ +	+ +	+
	Pt.05	Yes	49.0 (71.1%)	39.3	37.5	64.3	+ +	+	–	–
	Pt.06		128.0 (24.5%)	0.0	0.0	0.0	+ +	+ + +	+	+ +
	Pt.07		31.7 (81.3%)	34.0	48.9	34.0	+ + +	+ + +	+	+
	Pt.08		133.2 (0%)	NA	NA	NA	–	NA	–	–
	Pt.09		95.6 (27.7%)	NA	NA	NA	+ + +	+ + +	+ +	+ +
	Pt.10		101.7 (23.1%)	NA	NA	NA	+ + +	+ +	–	–
	Pt.11		69.3 (47.5%)	7.6	15.2	4.5	+	–	+ +	–
	Pt.12	Yes	7.0 (94.7%)	100.0	100.0	85.7	–	–	+ +	–
	Pt.13	Yes	73.8 (44.1%)	0.0	4.1	0.0	+ +	–	+ +	+ + +
	Mean		**73.9 (50.4%)**	**28.0**	**37.1**	**39.4**				
Late- POLG disease	Pt.14		127.7 (1.3%)	0.0	8.5	0.0	+ + +	–	+	–
	Pt.15	Yes	23.4 (81.9%)	0.0	57.7	0.0	–	–	+	–
	Pt.16	Yes	66.8 (48.4%)	2.0	0.0	0.0	–	–	+	+ +
	Pt.17		107.6 (16.9%)	5.4	5.4	0.0	+	+	+	+ +
	Pt.18		87.9 (32%)	0.0	0.0	0.0	–	+	+ +	+
	Mean		**82.7 (36.1%)**	**1.5**	**14.3**	**0**				
mtDNA disease	Pt.19	Yes	73.5 (43.2%)	7.7	1.9	0.0	+ +	+	+	–
	Pt.20		125.1 (3.4%)	1.9	1.9	0.0	+ +	–	+	–
	Pt.21		96.8 (25.2%)	3.0	0.0	0.0	+ +	+	+ +	–
	Pt.22		72.3 (44.1%)	26.3	0.0	7	+	+	–	–
	Pt.23		60.2 (53.4%)	6.7	0.0	2.2	–	+	–	+
	Pt.24		82.8 (36%)	1.5	1.5	0.0	–	–	+	+
	Pt.25		85.6 (33.8%)	0.0	0	0.0	+	–	+	+ +
	Pt.26	Yes	54.3 (58%)	14.3	10.2	6.1	–	–	–	+ +
	Pt.27		123.4 (4.6%)	0.0	1.8	0.0	+	–	+	–
	Pt.28		91.8 (29%)	0.0	0.0	0.0	+ +	–	+ +	–
	Mean		**86.6 (33.1%)**	**6.1**	**1.7**	**1.5**				

A summary of the quantitative data (Purkinje cell density and percentage Purkinje cell OXPHOS protein deficiencies) and qualitative (LC3B, BNIP3, p62, c-Fos) neuropathological findings in paediatric and adult mitochondrial disease patient cerebellar tissues. Mean values per PMD group are presented in bold for the quantitative data

*Focal necrotic lesions identified in the post-mortem cerebellum blocks included in this study. Qualitative scoring system to assess the density and intensity of immunoreactivity of LC3B, BNIP3, p62, and c-Fos in PMD patient tissues in comparison to age, sex, and formalin fixation-matched control tissues: – (no difference in comparison to control tissues or no evidence of immunoreactivity); + (mild increase); +  + (moderate increase); +  +  + (severe increase)

Comparison of PMD patient tissues revealed a striking increase in GFAP and HLA-DR immunoreactivity localised to the focal lesions in tissues from the patients with Alpers’ syndrome (Fig. 2, Supplementary Fig. 4). Neighbouring cortical regions demonstrated a high abundance of HLA-DR + microglia, albeit astrocyte densities appeared normal (Supplementary Fig. 4). However, interestingly, the focal lesions in tissues from adult patients with late-POLG disease and mtDNA disease were not as clearly defined by gliosis (Supplementary Fig. 5). Furthermore, the focal lesions in tissues from patients with Alpers’ syndrome were characterised by a total loss of Purkinje cells. However, often some, albeit few, Purkinje cells were observed in focal lesioned cortex from adult patients with late-POLG disease and mtDNA disease, suggesting less severe degeneration and inflammatory pathology within these late-onset lesions.

### Increased c-Fos immunoreactivity

In response to a severe degeneration of inhibitory Purkinje cells and focal necrotic lesions in the cerebellar cortex of patients with PMD, we next investigated whether c-Fos protein expression, a marker of neuronal hyperactivity [9], was increased. In cerebellar tissues from control cases, minimal c-Fos immunoreactivity was observed suggesting a low basal expression of c-Fos (Supplementary Fig. 6). However, 7 of 13 patients with Alpers’ syndrome, 3 of 5 patients with late-POLG disease, and 4 of 10 patients with mtDNA disease showed detectable levels of c-Fos, in total constituting 50% of the patient cohort (Table 3, Supplementary Fig. 6), suggesting recent neuronal hyperactivity. Increased c-Fos protein expression was often localised to Purkinje cells within non-lesioned cortex (Supplementary Fig. 6); however, some patient tissues also demonstrated c-Fos labelling in demarcated regions of the granular cell layer and molecular cell layer (Fig. 2). Interestingly, the patient with the most striking pattern of c-Fos immunoreactivity (Fig. 2), which was observed in focal regions of the superior and inferior cerebellar lobes, was Patient 13, a young adult with early onset POLG disease who died during *status epilepticus*. These findings suggest hyperexcitability of the cerebellar cortex in patients with PMD.

### Mitochondrial OXPHOS protein deficiencies in Purkinje neurons

**Fig. 3:**
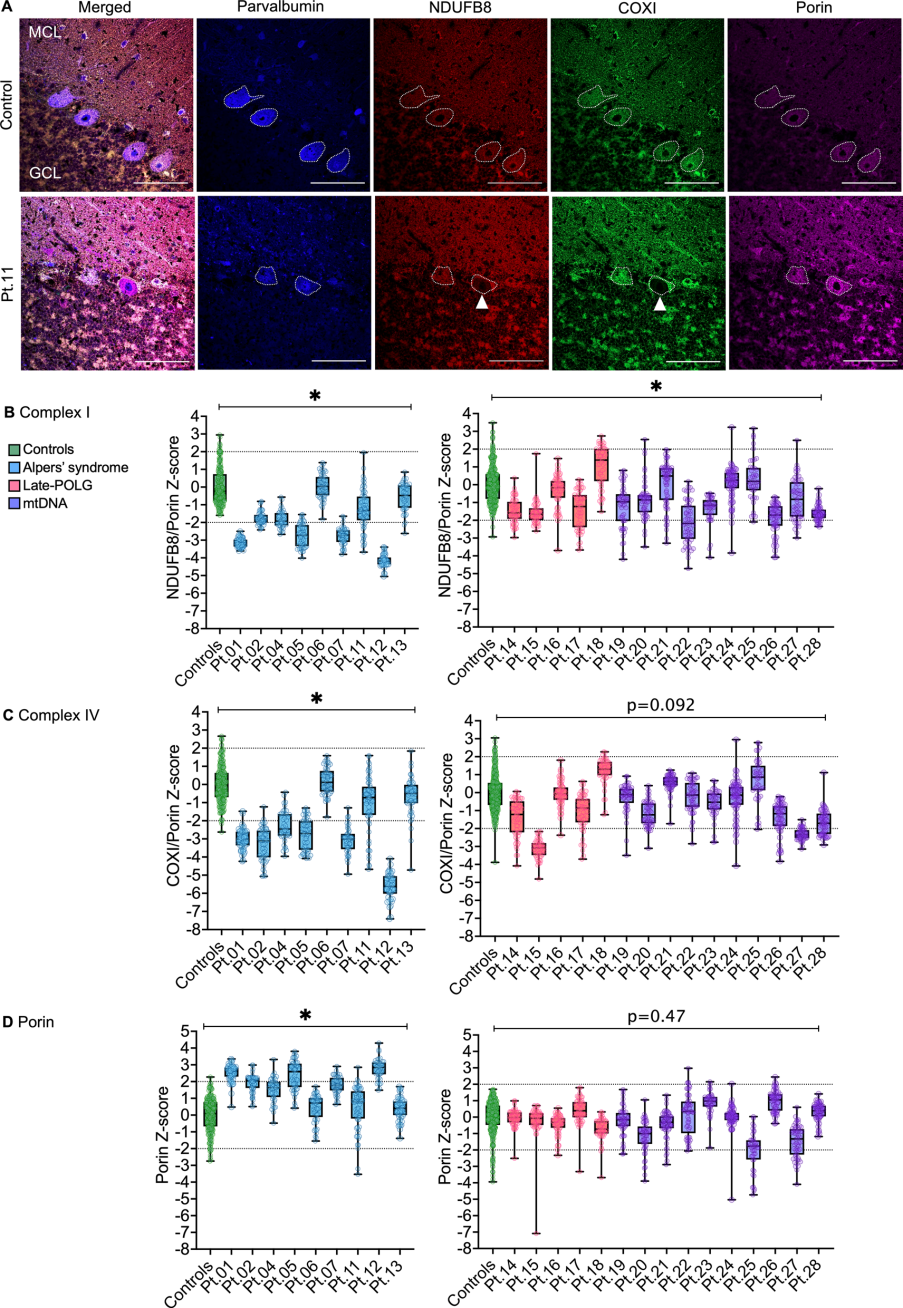
Mitochondrial oxidative phosphorylation protein deficiencies in Purkinje cells in mitochondrial disease. **a** Representative confocal images demonstrating quadruple immunofluorescence for parvalbumin (blue; Purkinje cell marker), NDUFB8 (red; complex I subunit), COXI (green; complex IV subunit), and porin (purple; mitochondrial mass marker) within the cerebellar cortex. Purkinje cell from Patient 11 (white arrow head) demonstrates decreased NDUFB8 and COXI immunoreactivity in comparison to adjacent Purkinje cell. **b** NDUFB8/Porin z-scores for individual Purkinje cells are presented for patients with Alpers’ syndrome (blue), adult-onset POLG (pink), and mtDNA disease (purple). Complex I deficiencies were significant in Alpers’ syndrome and adult-onset mitochondrial disease compared to age-matched control groups (*P* = 0.0179 and 0.0281, respectively, linear mixed-effects model). **c** COXI/Porin z-scores for Purkinje cells were significantly decreased in the Alpers’ syndrome group and did not reach significance in the adult mitochondrial disease group (*P* = 0.023 and 0.0918, respectively, linear mixed-effects model). (**d**) Porin z-scores were significantly increased in the Alpers’ syndrome group and remained unaltered in the adult mitochondrial disease group (*P* = 0.0122 and 0.4736, respectively, linear mixed-effects model). *P < 0.05

Since we quantified a severely decreased density of Purkinje cells in PMD patient tissues, we sought to investigate the vulnerability of this cell type to mitochondrial OXPHOS protein deficiencies to infer changes to OXPHOS function. Previously, OXPHOS protein defects have been reported in Alpers’ syndrome and late-onset PMD [4, 8]; however, comparative analyses have not been performed. Therefore, a quadruple immunofluorescence assay was used to quantitatively assess and compare the level of complex I (NDUFB8) and complex IV (COXI) subunit protein expression within Purkinje cells of PMD and control tissues (Fig. 3A).

Quantification of the intensity of NDUFB8 and COXI proteins, normalised to porin, within Purkinje neurons revealed OXPHOS protein deficiencies involving both complexes in the Alpers’ syndrome patient group relative to matched controls (*P* < 0.05) (Fig. 3B and C). However, in the late-POLG and mtDNA PMD cohort, only NDUFB8 protein expression was significantly decreased (*P* = 0.0281). Furthermore, comparison of the three PMD groups revealed that the Alpers’ syndrome patients demonstrated significantly lower expression of COXI in comparison to the mtDNA disease group (*P* = 0.0406). A summary of the percentage of neurons deficient for complex I and IV and Purkinje cell loss is provided in Table 3, which highlights the increased severity of changes to Purkinje cells in Alpers’ syndrome in comparison to late-POLG and mtDNA PMD groups (Supplementary Table 7).

To assess changes to mitochondrial mass within Purkinje neurons, the intensity of porin was quantified. Analyses revealed that porin was significantly increased in Alpers’ syndrome patient Purkinje cells relative to the control, late-POLG and mtDNA groups (*P* < 0.05), suggestive of increased mitochondrial mass. In contrast, porin remained unaltered in the late-POLG and mtDNA group relative to controls (Fig. 3D).

Finally, we quantified the expression of the activity-dependent calcium-binding protein parvalbumin (Supplementary Fig. 7) [34]. A lower expression of parvalbumin was detected within Purkinje neurons in the late-POLG and mtDNA disease groups (*P* = 0.0139); however, parvalbumin expression was unaltered at the Alpers’ syndrome patient group level (*P* = 0.0564).

### OXPHOS protein expression in the granule cell layer and dentate nucleus neurons

Since the expression of mitochondrial OXPHOS proteins has not previously been interrogated in neurons of the granule cell layer of patients with PMD, we sought to quantify OXPHOS protein expression within this predominantly excitatory cortical layer, as a comparison to inhibitory Purkinje cells (Supplementary Fig. 8A).

In Alpers’ syndrome, both NDUFB8 and COXI were significantly decreased in the granule cell layer relative to controls (*P* < 0.05), whereas in the late-POLG and mtDNA group, only COXI protein expression was significantly decreased (Supplementary Fig. 8B and C). Analyses between the three disease groups revealed a significantly increased NDUFB8 and COXI deficiencies in Alpers’ syndrome in comparison to the mtDNA and late-POLG disease groups (*P* < 0.01, Supplementary Table 7). Mitochondrial mass within the granular cell layer was significantly higher in patients with Alpers’ syndrome compared to controls (*P* = 0.0344), whereas mitochondrial mass remained unaltered in the late-POLG and mtDNA disease patient groups (Supplementary Fig. 8D). Interestingly, the level of OXPHOS protein deficiencies was similar between Purkinje neurons and the granule cell layer in patients with Alpers’ syndrome. However, the late-POLG and mtDNA patient groups demonstrated a more pronounced decrease in NDUFB8 and COXI expression in Purkinje neurons in comparison to the granule cell layer.

Additionally, we probed OXPHOS subunit expression in neurons of the dentate nucleus, as they are innervated by Purkinje cells and are primarily composed of excitatory neuronal populations. Intriguingly, dentate nucleus neurons did not demonstrate significantly altered NDUFB8, COXI, or porin protein levels in the PMD patient tissues relative to control tissues (*P* > 0.05, Supplementary Fig. 9). However, there was a significant decrease in the surface area of the dentate nucleus neurons, indicative of atrophy, in the Alpers’ syndrome and mtDNA groups (*P* < 0.05, Supplementary Table 7).

### Altered expression of autophagy-related proteins

Since OXPHOS-deficient Purkinje cells from patients with Alpers’ syndrome demonstrated increased porin abundance, indicative of increased mitochondrial mass, we sought to investigate whether this might be associated with impairments to mitophagy, the selective autophagic process whereby dysfunctional mitochondria are degraded [33]. We therefore probed the expression of mitophagy- and autophagy-related proteins, which are involved in the formation of mitophagosomes, semi-quantitatively in the PMD and control cerebellar tissues (Table 3). The markers employed included BNIP3 (mitophagy receptor), LC3B (marker of autophagosome formation), p62 (cargo autophagy receptor), and LAMP2 (lysosomal membrane protein).

**Fig. 4:**
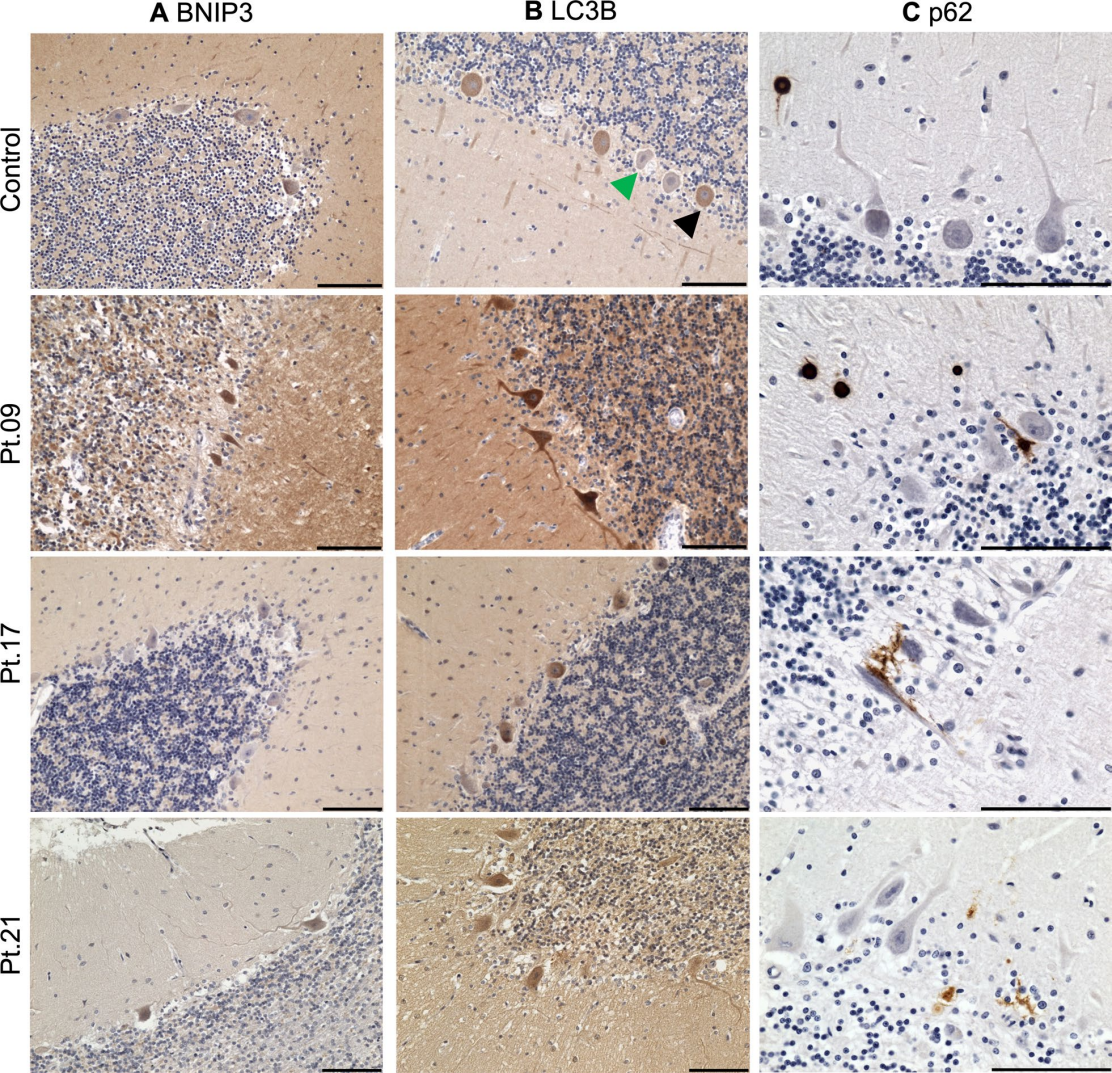
Increased expression of autophagy and mitophagy-related proteins in the cerebellar cortex in mitochondrial disease. Representative images demonstrate immunoreactivity of the mitophagy receptor BNIP3, autophagosome membrane marker LC3B, and autophagy receptor p62 in control tissue, as well as in patients with Alpers’ syndrome (Patient 9), late-POLG (Patient 17), and m.3243A > G MELAS syndrome (Patient 21). BNIP3 expression was increased in patients with Alpers’ syndrome, relative to controls, late-POLG, and mtDNA cases. LC3B protein expression was detected in a subset of Purkinje cells in control tissues (black arrowhead), in comparison to cells with undetectable levels of LC3B (green arrowhead). Most PMD patients demonstrated increased expression of LC3B relative to control cases. P62 immunoreactivity was observed primarily in corpora amylacea, some molecular layer inhibitory interneurons and vessels in adult controls and patients with PMD. However patient tissues demonstrated some p62-expressing glial cells, which appear to morphologically resemble microglia. All sections were counterstained with Haematoxylin. Scale bars = 100 μm

In cerebellar tissues from patients with Alpers’ syndrome, remaining Purkinje cells demonstrated a marked increased expression of both BNIP3 and LC3B proteins, compared to controls and patients with late-POLG and mtDNA disease (Fig. 4), mirroring the more extensive and severe OXPHOS deficiencies also observed in this patient group. A similar, albeit less pronounced, visibly increased expression of LC3B was observed in Purkinje cells from adult patients with late-POLG and mtDNA disease, whilst BNIP3 expression was only mildly increased in some of these cases (Table 3). However, LAMP2 protein expression appeared to be similar across all control and PMD cases (Supplementary Fig. 10).

P62-immunoreactive corpora amylacea and inhibitory interneurons within the molecular layer of the cerebellum were detected in most adult PMD patient tissues, and to a lesser extent in adult controls (Fig. 4). Infant controls unsurprisingly did not demonstrate any p62 corpora amylacea, since these structures are typically associated with degenerative and ageing processes, albeit p62 immunoreactivity was evident in multiple young patients with Alpers’ syndrome (Table 3). In some PMD patient tissues, p62-expressing glial cells were observed in close proximity to Purkinje neurons; however, the majority of patient tissues did not demonstrate any p62 immunoreactivity within the remaining Purkinje cells. An exception to this was Patient 18, in whose tissue intranuclear p62-immunoreactive puncta were present in virtually all Purkinje neurons.

Overall, we found an increased expression of autophagy-related proteins in PMD patient cerebellar tissues. However, the most prominent changes were detected in the Alpers’ syndrome patient tissues, which potentially reflects greater OXPHOS defects and increased mitochondrial mass in this disease group.

## Discussion

Degeneration of the cerebellar cortex is a frequent and often severe neuropathological feature of paediatric and adult PMD [4, 8, 19, 27, 31, 47]. The dysfunction and degeneration of inhibitory Purkinje cells is hypothesised to disrupt the cerebellar cortical circuitry and contribute to neurological manifestations including ataxia, which affected over a quarter of patients with PMD in our clinical cohort, and may be associated with stroke-like episodes. To better delineate the neuropathological mechanisms underlying cerebellar degeneration in paediatric and adult PMD, we performed a comparative, quantitative, neuropathological study examining the cerebellar cortex in a post-mortem tissue cohort from children and adults with PMD.

We have demonstrated a marked degeneration of Purkinje cells, accompanied by OXPHOS protein deficiencies, altered expression of key mitophagy-related proteins, and increased c-Fos protein expression suggestive of altered cerebellar activity. Overall, more severe neuropathological features were observed in the Alpers’ syndrome group, in comparison to the late-POLG and mtDNA disease groups, suggesting differential vulnerabilities of the cerebellar cortex between the subgroups of PMD. This may partly explain the typically more severe and rapidly progressive clinical presentation of early-onset PMD relative to late-onset PMD.

### Vulnerability of Purkinje cells to degeneration

Purkinje cells are critical inhibitory neurons responsible for the efferent output of the cerebellar cortex to inhibit the cerebellar nuclei, and thus have a fundamental role in balance control and movement coordination [25]. Moreover, recurring neuroimaging and neuropsychological findings have highlighted the intricate connectivity between the cerebellar and cerebral cortices, providing evidence that the cerebellum is also involved in cognitive function and behaviour [13, 17]. In line with the previous neuropathological studies [8, 19], we have demonstrated a severely decreased density of inhibitory Purkinje cells in cerebellar tissues of different subgroups of PMD. However, neuropathological features, including cortical atrophy, depletion of the granular cell layer, gliosis, and focal necrotic lesions, were more apparent and severe in the Alpers’ syndrome group. This suggests that there is an increased severity of cerebellar degeneration in childhood PMD compared to late-onset forms, which is likely to underpin the rapid progression and increased severity of childhood PMD.

Purkinje neurons have greater energy demands than granule cells in the cerebellar cortex as they have a larger cell surface area [12], and higher rates of firing [48]. Therefore, we hypothesised that this cell type would be particularly vulnerable to mitochondrial dysfunction in comparison to smaller, predominantly excitatory cells of the granule cell layer. Our data demonstrated that patients with Alpers’ syndrome harboured more severe OXPHOS protein deficiencies in comparison to patients with late-POLG and mtDNA disease, which may be associated with the increased degeneration of the cerebellar cortex in Alpers’ syndrome. Interestingly, our data also revealed that in adult patients with PMD, there appears to a hierarchy of vulnerability to OXPHOS deficiencies across neuronal populations, with Purkinje neurons demonstrating the most severe deficiencies, followed by granule cells and dentate nucleus neurons, which are both predominantly excitatory neurons. Thus, this observation corroborates our hypothesis that in adult PMD, there is a predilection for large inhibitory neurons to display more severe OXPHOS defects in comparison to smaller excitatory neurons in the cerebellum. However, in patients with Alpers’ syndrome, OXPHOS deficiencies in Purkinje neurons and granule cells were comparatively severe, perhaps providing an explanation for the more severe phenotype and neuropathological changes associated with this disorder.

### Altered autophagic demand in primary mitochondrial disease

Intriguingly, the disparities in OXPHOS protein deficiencies between the Alpers’ syndrome and adult PMD patient groups were mirrored by an increased expression of the autophagy- and mitophagy-related proteins LC3B and BNIP3 within Purkinje cells of patients with Alpers’ syndrome. However, it remains unclear whether the apparent increased expression of these markers indicates an increased rate of mitophagy, or alternatively, is reflecting a cessation or impairment of autophagy-related pathways. The latter may in part explain the increased mitochondrial mass observed in Purkinje neurons in patients with Alpers’ syndrome. In this post-mortem tissue cohort, increased BNIP3 expression in Purkinje neurons was limited to patients with Alpers’ syndrome and was not detected in most tissues from patients with late-POLG or mtDNA disease. Since activation of the BNIP3/NIX pathway is known to induce cell death [51], we may speculate that due to the heightened severity of mitochondrial dysfunction in Alpers’ syndrome, BNIP3 may be upregulated both to induce mitophagy, and may contribute to the activation of cell death pathways preceding neuronal attrition. However, we recognise the limitations of immunohistochemically assessing autophagy, a dynamic process, in post-mortem tissues; thus, these pathways should be interrogated in functional patient derived in vitro models using autophagy flux assays [16].

### Focal necrotic lesions and evidence of cerebellar hyperactivity in mitochondrial disease

Stroke-like episodes in PMD are characterised by an acute, or subacute, onset of focal seizures, encephalopathy and focal neurological deficits, which are hypothesised to be driven by neuronal hyperexcitability, and are not caused by arterial thrombo-embolism [14, 30]. Progressive brain atrophy, including the cerebellum, is frequently observed following these clinical events in adult patients with PMD [31]. In the current study, we identified demarcated focal necrotic lesions in cerebellar cortices which were more severe in the Alpers’ syndrome patient group, compared to patients with late-POLG and mtDNA diseases. The focal necrotic lesions observed in tissues from the younger patients typically affected all cerebellar cortical layers and could be clearly identified by focal astrogliosis and microgliosis, processes that were less evident in lesions from patients with late-POLG and mtDNA disease. This observation may potentially indicate a more severe level of neuroinflammatory changes in Alpers’ syndrome and may be an important contributor to the more severe clinical presentation of paediatric-onset PMD in comparison to late-onset PMD.

Our novel observation of very low parvalbumin immunoreactivity localised within focal necrotic lesions was a shared feature across all PMD patient groups. We therefore propose that this offers value in using anti-parvalbumin antibodies for rapid identification of lesions immunohistochemically. Decreased parvalbumin immunoreactivity may be due to a combination of decreased density, or absence, of Purkinje cells and parvalbumin molecular cell layer interneurons, in addition to a downregulation of parvalbumin protein within remaining cells due to decreased activity resulting from mitochondrial dysfunction.

In the post-mortem tissue cohort, we also identified “intermediate” lesioned cortex which had lower parvalbumin immunoreactivity and intermediate level of HLA-DR-immunoreactive microglial cells. However, GFAP immunoreactivity appeared relatively normal, which leads us to speculate that microglial activation may potentially precede astrogliosis at earlier stages of necrotic lesion formation. However, this warrants further investigation using in vivo and in vitro model systems of mitochondrial disease pathology.

The widespread degeneration and dysfunction of Purkinje cells, accompanied by the striking absence of Purkinje cells within focal necrotic lesions, will undoubtedly contribute to cerebellar cortical hyperexcitability and may explain the increased c-Fos protein expression observed in many patient tissues. In patients with Alpers’ syndrome, increased protein expression of c-Fos, a gene transiently expressed as an early response to neuronal activity [9], was observed relative to patients with late-POLG and mtDNA PMD, indicating hyperexcitability in subregions of the cerebellar cortex. Increased c-Fos protein expression was identified in multiple cell types across different cerebellar cortical layers which may indicate a widespread dysregulated, excitable cerebellar circuit. This is hypothesised to be a consequence of impaired firing and degeneration of Purkinje cells [24, 26], resulting in aberrant firing of deep cerebellar nuclei neurons [41]. Interestingly, we did not identify focal necrotic cerebellar lesions in adult patients who did not have epilepsy. Since the cerebellum has been implicated in the pathogenesis of seizures [41, 45], and epilepsy in early-onset POLG-related disease is often more refractory compared to late-onset mitochondrial epilepsy [22], it is possible that there is an association between cortical and cerebellar hyperexcitability, focal necrotic cerebellar lesions, and epilepsy in PMD. However, this observation warrants further investigations in larger PMD patient cohorts, with and without epilepsy.

We previously reported a decrease in parvalbumin protein expression in parvalbumin-expressing cortical interneurons in PMD patient tissues [32, 43]. These findings were corroborated in the remaining Purkinje neurons in patients with late-POLG and mtDNA disease, which may potentially indicate a reduced rate of firing of the remaining Purkinje cells. Interestingly, there is evidence suggesting that decreased parvalbumin expression modulates and upregulates the expression of mitochondrial biogenesis genes which encode for PGC1α, TFAM, and NRF1 [3, 21]. One possible explanation to this phenomenon is to support Ca^2+^ buffering via increased mitochondrial volume to compensate for the loss of Ca^2+^-buffering parvalbumin protein. However, despite this, we did not observe an increase in mitochondrial mass in Purkinje neurons in adult patients with PMD, as the increase in porin expression was only detected in the Alpers’ syndrome patient group. An increase in porin may reflect an adaptive mitochondrial biogenesis induction in response to OXPHOS deficiencies and/or reflect stalled mitophagy which may fail to degrade dysfunctional mitochondria. Interestingly, overexpression of porin can be induced by increased intracellular Ca^2+^ concentration which has also been shown to induce apoptosis in multiple cell types [42]. Increased porin expression in Purkinje neurons in cerebellar tissues from patients with Alpers’ syndrome coupled with enhanced c-Fos immunoreactivity, which has also been reported in patient primary visual cortex tissues [43], may indicate aberrant Ca^2+^ accumulation and excitotoxicity.

### Limitations of this study

Whilst human post-mortem tissues represent an invaluable resource for delineating cellular and molecular changes associated with PMD, the neurodegenerative features identified in this study represent end-stage pathologies. These findings will, however, be crucial for assessing whether in vitro and in vivo model systems fully recapitulate the pathological features presented by patients with PMD. Although, to date, this remains the largest post-mortem PMD neuropathological study, due to the rarity of Alpers’ syndrome, we included historical post-mortem tissues from patients that preceded the genetic discovery of *POLG-*related PMD. Furthermore, due to differences in brain dissection protocols, unfortunately, we were unable to perform detailed anatomical assessment and determine any selective vulnerability in the connections between the cerebrum and cerebellum. However, it was noticeable that both the superior and inferior cerebellar lobes were affected across the patient cohort. This also precluded assessment of the dentate nucleus for all control and patient cases.

## Conclusions

Our post-mortem neuropathological study has demonstrated a severe vulnerability of inhibitory Purkinje cells to mitochondrial dysfunction and degeneration in paediatric and adult patients with POLG and mtDNA PMDs. The dysfunction and depletion of Purkinje cells is hypothesised to impair inhibitory regulation of the cerebellar nuclei, which may promote the development of a hyperexcitable cerebellar cortical circuit. Whilst we speculate that this is an important contributor to the development of ataxia in patients with PMD, further studies are warranted to address this functionally. Furthermore, this impairment of inhibitory regulation may increase the propensity for seizure-driven stroke-like episodes and cross-cerebellar diaschisis, both of which may further exacerbate the dysregulation of the cerebellar circuitry. Importantly, our study highlights a more severe neuropathological profile in patients with early-onset POLG-related disease in comparison to late-POLG and mtDNA PMDs, which we hypothesise may partly underpin the severe and relentless clinical presentation of early-onset POLG-related disease.

## Supplementary Information

Below is the link to the electronic supplementary material.

Supplementary file1 (DOCX 17217 KB)

## Data Availability

The data supporting the findings of this study are available from the corresponding authors upon reasonable request.
