# Supplementary material for: Delineating the mechanisms of cerebellar degeneration in paediatric and adult primary mitochondrial disease
Source: Acta Neuropathol. 2025 May 30;149(1):53. doi: 10.1007/s00401-025-02891-6 (PMC12125081; doi:10.1007/s00401-025-02891-6)
Supplement: Supplementary file 1 — Supplementary file1 (DOCX 17217 KB) [file 401_2025_2891_MOESM1_ESM.docx]

**Supplementary Information:**

**Delineating the mechanisms of cerebellar degeneration in paediatric and adult primary mitochondrial disease**

**Authors**: Laura A. Smith^1,†^ and Elizaveta A. Olkhova^1,2^,^†^, Nichola Z. Lax,^1^ Yi Shiau Ng^3,4,5^, Robert W. Taylor^1,3^, Grainne S. Gorman^3,4,6^, Daniel Erskine^1^, Robert McFarland^1,3^

^†^ These authors contributed equally to this work.

**Author affiliations**:

^1^ Mitochondrial Research Group, Translational and Clinical Research Institute, Faculty of Medical Sciences, Newcastle University, Newcastle upon Tyne, NE2 4HH, UK.

^2^ Translational Biosciences, Applied Sciences, Ellison Building, Northumbria University, Newcastle upon Tyne, NE1 8ST, UK.

^3^ NHS Highly Specialised Service for Rare Mitochondrial Disorders of Adults and Children, Newcastle upon Tyne Hospitals NHS Foundation Trust, Newcastle Upon Tyne, NE1 4LP, UK.

^4^ National Institute for Health and Care Research (NIHR), Newcastle Biomedical Research Centre (BRC), Newcastle University, Newcastle upon Tyne, NE4 5PL.

^5^ Directorate of Neurosciences, Royal Victoria Infirmary, Newcastle upon Tyne Hospitals NHS Foundation Trust, Newcastle upon Tyne, NE1 4LP.

^6^ Wellcome Centre for Mitochondrial Research. Translational and Clinical Research Institute. Faculty of Medical Sciences, Newcastle University, Newcastle upon Tyne, NE2 4HH, UK.

**Corresponding authors:**

Professor Robert McFarland: robert.mcFarland@newcastle.ac.uk

Dr Laura Smith: laura-alexandra.smith@newcastle.ac.uk

Mitochondrial Research Group, Translational and Clinical Research Institute, Faculty of Medical Sciences, Newcastle University, Newcastle upon Tyne, NE2 4HH, UK

**Supplementary information**:

**Immunohistochemistry protocol**:

Briefly, sections were deparaffinised, rehydrated and subjected to antigen retrieval in either 10 mM citrate pH 6.0 or 1 mM EDTA pH 8.0. Sections were then blocked with 3% hydrogen peroxide (H_2_O_2_) and primary antibodies were optimally diluted and applied overnight at 4°C (**Supplementary Table 3**). The Menarini Diagnostics horseradish peroxidase polymer kit was used for primary antibody amplification and protein targets were visualised using 3,3’-diaminobenzidine (DAB) chromogen. Sections were counterstained using Mayer’s haematoxylin and Scott’s Tap Water to visualise nuclei.

**Immunofluorescence protocol**:

Five µm-thick FFPE sections were deparaffinised, rehydrated and subjected to antigen retrieval in 1 mM EDTA pH 8.0, and were blocked with 10% normal goat serum (NGS) in Tris-buffered saline solution containing 0.05% Tween 20 (TBST). Primary antibodies were then optimally diluted and applied overnight at 4°C (**Supplementary Table 3**). To amplify the primary antibody against NDUFB8, a biotinylated goat anti-mouse IgG1 antibody was applied to the sections for 30 minutes at room temperature, prior to incubation with an AlexaFluor-conjugated secondary antibody cocktail for two hours at 4°C (**Supplementary Table 4**). Following this, autofluorescence was quenched using 3% Sudan Black B solution and sections were then mounted using Prolong Gold. Single stained sections and a no-primary antibody section were included in the staining batch to control for bleed-through between channels and background correction, respectively.

**Supplementary Table 1** Mitochondrial Disease Patient Clinical Cohort

| **Genotype** | **Number of patients** | | **Age of patients (years)** | | **Interval between assessments** | **Sex (Female: Male)** | |
| --- | --- | --- | --- | --- | --- | --- | --- |
|  | *First assessment* | *Most recent assessment* | *First assessment* | *Most recent assessment* |  | *First assessment* | *Most recent assessment* |
| **m.13094T>C** | 3 | 0 | 33.67 | NA | NA | 3:0 | NA |
| **m.14709T>C** | 8 | 6 | 47.63 | 56.50 | 14.67 | 8:0 | 6:0 |
| **m.3243A>G** | 345 | 248 | 40.72 | 46.54 | 6.12 | 204:141 | 146:102 |
| **m.8344A>G** | 51 | 34 | 41.02 | 45.65 | 5.26 | 31:20 | 21:13 |
| ***POLG*** | 32 | 22 | 41.44 | 52.09 | 5.64 | 19:13 | 13:9 |
| **All genotypes** | **439** | **310** | **41.01** | **47.03** | **6.2** | **265:174** | **186:124** |

Summary of the MRC Mitochondrial Disease Patient Cohort. Mean values are presented for the age of patients (years) and interval between assessments (years). *NA*; not assessed.

Only patients with follow-up assessments (n=310) were included for further analyses. Median interval was 5 years, and mean interval was 6.2 years between the first and most recent follow up assessment.

| **Supplementary Table 2.** Mitochondrial disease patient post-mortem tissue cohort | | | | | | | | | | |
| --- | --- | --- | --- | --- | --- | --- | --- | --- | --- | --- |
| **Case** | **Brain bank** | **Clinical diagnosis** | **Molecular genetics** | | **Sex** | **Age at death** | **PMI** | **Formalin fixation** | **Cause of death** | **Previous publications** |
|  |  |  | **Protein** | **DNA** |  |  |  |  |  |  |
| Pt.01 | NBTR | Alpers’ syndrome | p.[Ala467Thr]/ p.[Gly848Ser] | c.1399G>A/ c.2542G>A | F | 14 m | 12 h | 1 m | Hepatic failure | Hayhurst et al., 2018. Smith et al., 2022. Smith et al., 2023. |
| Pt.02 | Brain UK (Southampton) | Alpers’ syndrome | Unknown | Unknown | M | 17 m | Unknown | 6 m | Unknown | Smith et al., 2022. Smith et al., 2023. |
| Pt.03 | NBB | Alpers’ syndrome | Unknown | Unknown | F | 18 m | 17 h | 18.8 y | Respiratory failure | Smith et al., 2022. Smith et al., 2023. |
| Pt.04 | Brain UK (Oxford) | Alpers’ syndrome | Unknown | Unknown | M | 2.8 y | Unknown | 4.0 m | Respiratory failure | Smith et al., 2022. Smith et al., 2023. |
| Pt.05 | NBTR | Alpers’ syndrome | p.[Cys418Arg]/p.[Ala467Thr] | c.1252T>C/ c.1399G>A | M | 3.2 y | 18 h | 3.0 m | Multi-organ failure | No previous publications. |
| Pt.06 | Brain UK (Bristol) | Alpers’ syndrome | Unknown | Unknown | F | 4 y | 24 h | 3.0 m | Respiratory failure | Hayhurst et al., 2018. Smith et al., 2022. Smith et al., 2023. |
| Pt.07 | Vienna | Alpers’ syndrome | p.[Trp748Ser]/ p.[Trp748Ser] | c.2243G>C/ c.2243G>C | F | 7 y | Unknown | Unknown | Pneumonia | Hayhurst et al., 2018. Smith et al., 2022. Smith et al., 2023. Jellinger et al., 1970. |
| Pt.08 | NBB | Alpers’ syndrome | p.[Ala467Thr]/ p.[Gly848Ser] | c.1399G>A/ c.2542G>A | M | 11.9 | 21 h | 9 y | Complication of disorder | Smith et al., 2022. Smith et al., 2023. |
| Pt.09 | Oxford Brain Bank | Alpers’ syndrome | Unknown | Unknown | M | 12.5 | Unknown | 32 y | Uncontrollable myoclonic epilepsy | Hayhurst et al., 2018. Smith et al., 2022. Smith et al., 2023. |
| Pt.10 | Oxford Brain Bank | Alpers’ syndrome | Unknown | Unknown | F | 14 y | 48 h | 17 y | Unknown | Hayhurst et al., 2018. Smith et al., 2022. Smith et al., 2023. |
| Pt.11 | NBTR | Alpers’ syndrome | p.[Ala467Thr]/ p.[Ala467Thr] | c.1399G>A/ c.1399G>A | F | 23 y | 32 h | 1 m | Status epilepticus | Ng et al., 2022. Smith et al., 2022. Smith et al., 2023. Flower et al., 2015. |
| Pt.12 | NBTR | Alpers’ syndrome | p.[Ala467Thr]/ p.[Trp748Ser] | c.1399G>A/ c.2243G>C | F | 24 y | 83 h | 4.5 m | Suppurative tracheobronchitis | Lax et al., 2016. Lax et al., 2012.A. Lax et al., 2012.B. Lax et al., 2012.C. Ng et al., 2022. Smith et al., 2022. Smith et al., 2023. Chan et al., 2019. |
| Pt.13 | NBTR | Alpers’ syndrome | p.[Ala467Thr]/ p.[Trp748Ser] | c.1399G>A/ c.2243G>C | F | 28 y | 64 h | 1 m | Status epilepticus | Ng et al., 2022. Smith et al., 2022. Smith et al., 2023. |
| Pt.14 | NBTR | Late-POLG | p.[Ala467Thr]/ p.[Ala467Thr] | c.1399G>A/ c.1399G>A | F | 46 y | 105 h | 1.5 m | Multi-organ failure | No previous publications. |
| Pt.15 | NBTR | Late-POLG | p.[Ala467Thr]/ p.[X1240Cys] | c.1399G>A/ c.3718T>C | M | 50 y | 20 h | 12 m | N/A | Lax et al., 2012.C. Ng et al., 2022. |
| Pt.16 | NBTR | Late-POLG | p.[Trp748Ser]/ p.[Arg1096Cys] | c.2243G>C/ c.3286C>T | M | 55 y | 112 | 2 m | Lower respiratory tract infection | Chrysostomou et al., 2016. Lax et al., 2016. Chan et al., 2019. Ng et al., 2022. Olkhova et al., 2023. |
| Pt.17 | NBTR | Late-POLG | p.[Gly848Ser]/ p.[Ser1104Cys] | c.2542G>A/ c.3311C>G | M | 59 y | 67 h | 2.2 m | Suppurative tracheobronchitis | Chrysostomou et al., 2016. Lax et al., 2012.A. Lax et al., 2012.C. Ng et al., 2022. Olkhova et al., 2023. |
| Pt.18 | NBTR | Late-POLG | p.[Thr251Ile]/ p.[Pro587Leu],  p.[Ala467Thr] | c.752C>T/ c.1760C>T, c.1399G>A | M | 79 y | 85 h | 2 m | Pneumonia | Chrysostomou et al., 2016. Chan et al, 2019. Lax et al., 2016. Ng et al., 2022. Olkhova et al., 2023. |
| Pt.19 | NBTR | MELAS |  | m.3243 A>G | F | 20 y | 187 h | 1.5 m | Aspiration pneumonia | Chrysostomou et al., 2016. Chan et al, 2019. Lax et al., 2012.A. Lax et al., 2012.B. Ng et al., 2022. |
| Pt.20 | NBTR | MELAS |  | m.3243 A>G | F | 42 y | < 24 h | 6 m | N/A | Lax et al., 2012.A. Lax et al., 2012.B. Lax et al al., 2016. Ng et al., 2022. |
| Pt.21 | NBTR | MELAS |  | m.3243 A>G | M | 45 y | 43 h | 2.2 m | N/A | Chrysostomou et al., 2016. Chan et al, 2019. Lax et al., 2012.A. Lax et al., 2012.B. Lax et al al., 2016. Ng et al., 2022. Olkhova et al., 2023. |
| Pt.22 | NBTR | MELAS |  | m.3243 A>G | F | 64 y | 103 h | 2 m | Terminal stage of MELAS | Ng et al., 2022. Olkhova et al., 2023. |
| Pt.23 | NBTR | MERRF |  | m.8344 A>G | M | 31 y | 51 h | 2 m | Respiratory failure | Olkhova et al., 2023. |
| Pt.24 | NBTR | MERRF |  | m.8344 A>G | F | 42 y | 59 h | 1 m | Respiratory failure | Chrysostomou et al., 2016. Lax et al., 2012.A. Lax et al., 2012.B. Lax et al al., 2016. Ng et al., 2022. Olkhova et al., 2023. |
| Pt.25 | NBTR | MERRF/ MELAS overlap |  | m.8344 A>G | M | 58 y | 66 h | 2 m | Stroke-like episodes | Chrysostomou et al., 2016. Chan et al, 2019. Lax et al., 2016. Lax et al., 2012.B. Ng et al., 2022. Olkhova et al., 2023. |
| Pt.26 | NBTR | MERRF |  | m.8344 A>G | F | 64 y | 42 h | 5 m | N/A | No previous publications. |
| Pt.27 | NBTR | MELAS/ Leigh Syndrome overlap |  | m.13094 T>C | F | 34 y | 28 h | 1.2 m | N/A | Lax et al., 2012.A. Lax et al., 2012.B. Ng et al., 2022. |
| Pt.28 | NBTR | Cognitive impairment, peripheral neuropathy, diabetes mellitus, CPEO, IHD, PVD |  | m.14709T>C | M | 55 y | 24 h | 4.5 m | Myocardial infarction | Chrysostomou et al., 2016. Lax et al., 2012.A. Lax et al., 2012.B. Ng et al., 2022. |

**Abbreviations**: *Pt* Patient; *NBTR* Newcastle Brain Tissue Resource; *NBB* NeuroBioBank; *m* months; *y* years; *h* hours; *PMI* post-mortem interval; *N/A* not available. *MERRF* Myoclonic epilepsy with ragged red fibres; *MELAS* Mitochondrial Encephalopathy, Lactic Acidosis and Stroke-like episodes; *CPEO* Chronic progressive external ophthalmoplegia; *IHD* ischaemic heart disease; *PVD* peripheral vascular disease.

RefSeq: NM_002693.3 and NC_012920.1. Historical patients lacking a molecular genetic diagnosis of Alpers’ syndrome precede identification that pathogenic variants in *POLG* cause Alpers’ syndrome and extraction of DNA from FFPE samples for *POLG* sequencing was unsuccessful.

**References for previous publications (Supplementary Table 2)**:

Chan F, Lax NZ, Voss CM, Aldana BI, Whyte S, Jenkins A, Nicholson C, Nichols S, Tilley E, Powell Z (2019) **The role of astrocytes in seizure generation: insights from a novel in vitro seizure model based on mitochondrial dysfunction**. Brain 0: 1-2

Chrysostomou A, Grady JP, Laude A, Taylor RW, Turnbull DM, Lax NZ (2016) **Investigating complex I deficiency in Purkinje cells and synapses in patients with mitochondrial disease**. Neuropathology and applied neurobiology 42: 477-492 Doi 10.1111/nan.12282

Flower M, Ali K, Lawthom C (2015) **Status epilepticus caused by an unusual encephalopathy**. Pract Neurol 15: 56-59 Doi 10.1136/practneurol-2014-000884.

Hayhurst H, Anagnostou, M. E., Bogle, H. J., Grady, J. P., Taylor, R. W., Bindoff, L. A., McFarland, R., Turnbull, D. M. & Lax, N. Z. (2018) **Dissecting The Neuronal Vulnerability Underpinning Alpers' Syndrome: A Clinical And Neuropathological Study**. Brain Pathology 29: 97-113.

Jellinger K, Seitelberger F (1970) **Spongy glio-neuronal dystrophy in infancy and childhood**. Acta Neuropathologica 16: 125-140 Doi 10.1007/BF00687667

Lax NZ, Grady J, Laude A, Chan F, Hepplewhite PD, Gorman G, Whittaker RG, Ng Y, Cunningham MO, Turnbull DM (2016) **Extensive respiratory chain defects in inhibitory interneurones in patients with mitochondrial disease**. Neuropathol Appl Neurobiol 42: 180-193 Doi 10.1111/nan.12238

Lax NZ, Hepplewhite PD, Reeve AK, Nesbitt V, McFarland R, Jaros E, Taylor RW, Turnbull DM (2012.A) **Cerebellar ataxia in patients with mitochondrial DNA disease: a molecular clinicopathological study**. J Neuropathol Exp Neurol 71: 148-161 Doi 10.1097/NEN.0b013e318244477d

Lax NZ, Pienaar IS, Reeve AK, Hepplewhite PD, Jaros E, Taylor RW, Kalaria RN, Turnbull DM (2012.B) **Microangiopathy in the cerebellum of patients with mitochondrial DNA disease**. Brain 135: 1736-1750 Doi 10.1093/brain/aws110

Lax NZ, Whittaker RG, Hepplewhite PD, Reeve AK, Blakely EL, Jaros E, Ince PG, Taylor RW, Fawcett PR, Turnbull DM (2012.C) **Sensory neuronopathy in patients harbouring recessive polymerase gamma mutations**. Brain 135: 62-71 Doi 10.1093/brain/awr326

Ng Y, Lax NZ, Blain A, Erskine D, Baker M, Polvikoski T, Thomas R, Morris C, Lai M, Whittaker Ret al (2022) **Forecasting stroke-like episodes and outcomes in mitochondrial disease**. Brain 145: 542-554

Olkhova EA, Bradshaw C, Blain A, Alvim D, Turnbull DM, LeBeau FEN, Ng YS, Gorman GS, Lax NZ (2023) **A novel mouse model of mitochondrial disease exhibits juvenile-onset severe neurological impairment due to parvalbumin cell mitochondrial dysfunction**. Communications Biology 6: 1078 Doi 10.1038/s42003-023-05238-7

Smith LA, Chen C, Lax NZ, Taylor RW, Erskine D, McFarland R (2023) **Astrocytic pathology in Alpers' syndrome**. Acta Neuropathol Commun 11: 86 Doi 10.1186/s40478-023-01579-w

Smith LA, Erskine D, Blain A, Taylor RW, McFarland R, Lax NZ (2022) **Delineating selective vulnerability of inhibitory interneurons in Alpers' syndrome**. Neuropathol Appl Neurobiol: e12833 Doi 10.1111/nan.12833

**Supplementary Table 3**. Control post-mortem tissue cohort

| **Case** | **Case ID** | **Brain Bank Source** | **Sex** | **Age at death** | **PMI** | **Formalin fixation duration** | **Cause of death** |
| --- | --- | --- | --- | --- | --- | --- | --- |
| Ct.01 | B4701 | Oxford | F | 7 m | Unknown | Unknown | Unknown |
| Ct.02 | NP142.2013 | Oxford | M | 11 m | 96 h | Unknown | Unknown |
| Ct.03 | 4395 | NBB | F | 14 m | 20 h | 3.1 y | Coarctation of aorta |
| Ct.04 | 5334 | NBB | M | 12.7 y | 15 h | 9.3 y | Hanging / suicide |
| Ct.05 | 5309 | NBB | F | 14.5 y | 8 h | 9 y | Streptococcal toxic shock syndrome |
| Ct.06 | NP137.2005 | Oxford | M | 16 y | 72 h | 1 m | Unascertained sudden death |
| Ct.07 | SD001.06 | EBTB | M | 16 y | 47 h | 4 d | Suspension by ligature |
| Ct.08 | NA2016.0862 | NBTR | F | 18 y | 81 h | 6 m | MDMA toxicity, cardiac arrest |
| Ct.09 | SD023.08 | EBTB | F | 24 y | 47 h | 9 d | Suspension by ligature |
| Ct.10 | NA2018.5911 | NBTR | M | 42 y | 24 h | 1.5 m | Respiratory failure, sepsis |
| Ct.11 | NA2014.1903 | NBTR | M | 52 y | 102 h | 2 m | Metastatic mesothelioma |
| Ct.12 | NA2009.0118 | NBTR | M | 55 y | 41 h | 3.5 m | Liver cancer |
| Ct.13 | NA2013.0028 | NBTR | M | 56 y | 100 h | 4 m | Cardiomyopathy |
| Ct.14 | NA2016.0440 | NBTR | F | 59 y | 34 h | 2.2 m | Multiorgan failure, ischaemic leg, PVD |
| Ct.15 | NA2017.4911 | NBTR | M | 59 y | 78 h | 2.2 m | Metastatic sigmoid cancer |
| Ct.16 | NA2013.0400 | NBTR | F | 65 y | 47 h | 2.2 m | Metastatic ovarian cancer, non-Hodgkin’s lymphoma |
| Ct.17 | NA2005.0087 | NBTR | M | 68 y | 54 h | 2 m | Bowel cancer |
| Ct.18 | NA2011.0837 | NBTR | M | 81 y | 43 h | 2.5 m | Pneumonia, infective endocarditis |

Abbreviations: *NBTR* Newcastle Brain Tissue Resource; *NBB* NeuroBioBank; *EBTB* Edinburgh Brain Tissue Bank; *m* months; *y* years; *h* hours; *d* days; *PMI* post-mortem interval; *PVD* peripheral vascular disease.

The Alpers’ syndrome patient group (Pt.01–Pt.13, median age: 7 years, age range: 14 months to 28 years) were compared to nine neurologically normal controls (Ct.01–Ct.09, median age: 14.5 years, age range: 7 months – 24 years), matched for age at death (*P* = 0.883, Mann-Whitney), sex distribution (*P* = 0.999, Fisher’s exact test), post-mortem interval (*P* = 0.371, *t*-test) and formalin fixation length (*P* = 0.547, Mann-Whitney).

Fifteen adult patients with late-onset POLG disease and mtDNA disease (Pt.14–Pt.28, median age: 50 years, age range: 20 years – 79 years) were matched for age (*P* = 0.0932, *t*-test), sex distribution (*P* = 0.389, Fisher’s exact test), post-mortem interval (*P* = 0.693, Mann-Whitney) and tissue fixation length (*P* = 0.512, Mann-Whitney) to eight neurologically normal controls (Ct.10–Ct.18, median age: 59 years, age range: 42 years – 81 years).

**Supplementary Table 4** Primary antibodies

| **Antibody** | **Host (isotype)** | **Chromogen dilution** | **Immunofluorescence dilution** | **Antigen retrieval** | **Antibody supplier** | **Catalogue number (RRID)** |
| --- | --- | --- | --- | --- | --- | --- |
| **Parvalbumin** | Rabbit (IgG) | 1:2000 | 1:500 | 1mM EDTA (pH 8.0) pressure cooker | Swant | PV27 **(**RRID:AB_2631173) |
| **NDUFB8** (complex I subunit) | Mouse (IgG1) | NA | 1:100 |  | Abcam | ab110242  (RRID:AB_10859122) |
| **COXI** (complex IV subunit) | Mouse (IgG2a) | NA | 1:200 |  | Abcam | ab14705 **(**RRID:AB_2084810) |
| **Voltage-dependent anion channel 1** (VDAC1) / Porin | Mouse (IgG2b) | NA | 1:200 |  | Abcam | ab14734 **(**RRID:AB_443084) |
| **BNIP3** (mitophagy receptor) | Rabbit  (IgG) | 1:2000 | NA |  | Abcam | ab109362 **(**RRID:AB_10864714) |
| **LC3B** (LC3; lysosomal marker) | Rabbit  (IgG) | 1:5000 | NA |  | Abcam | ab48394 **(**RRID:AB_881433) |
| **LAMP2** (lysosome-associated membrane protein 2) | Rabbit (IgG) | 1:4,000 | NA |  | Abcam | Ab199946 **(**RRID:AB_2940865) |
| **C-fos** (transient early immediate gene) | Rabbit (IgG) | 1:2000 | NA |  | Abcam | ab222699 **(**RRID:AB_2891049) |
| **p62** (mitophagy adaptor) | Mouse (IgG1) | 1:500 | NA | 10mM Trisodium citrate (pH 6.0) microwave | BD Biosciences | 610833 **(**RRID:AB_398152) |
| **Glial fibrillary acidic protein** (GFAP) | Rabbit  (IgG) | 1:15,000 | NA |  | DAKO | Z0334 **(**RRID:AB_10013382) |
| **HLA-DP,DQ,DR** (human leukocyte antigen) | Mouse (IgG1) | 1:1000 | NA |  | DAKO | M0775 **(**RRID:AB_2313661) |

**Supplementary Table 5** Secondary antibodies

| **Antibody** | **Reactivity** | **Dilution** | **Antibody supplier** | **Catalogue number**  **(RRID)** |
| --- | --- | --- | --- | --- |
| XX Goat Biotin | Mouse IgG1 | 1:200 (30 mins room temperature) | Thermo Fisher Scientific | A10519  (RRID:AB_2534028) |
| Alexa Fluor 405 | Rabbit IgG | 1:100 (2 hours, 4 degrees) | Thermo Fisher Scientific | A31556  (RRID:AB_221605) |
| Alexa Fluor 488 | Mouse IgG2a | 1:100 (2 hours, 4 degrees) | Thermo Fisher Scientific | A21131  (RRID:AB_2535771) |
| Alexa Fluor 546-streptavidin | Biotin | 1:100 (2 hours, 4 degrees) | Thermo Fisher Scientific | S11225  **(**RID:AB_2532130) |
| Alexa Fluor 647 | Mouse IgG2b | 1:100 (2 hours, 4 degrees) | Thermo Fisher Scientific | A21244  **(**RRID:AB_2535812) |

**Supplementary Table 6.** Cerebellar neuropathology

| **Case** | **Age** | **Neuropathology summary** | **Focal lesions** | **Cerebellar pathology previously reported** |
| --- | --- | --- | --- | --- |
| Pt.01 | 14 m | Mild Purkinje cell loss; mild Bergmann gliosis. |  | Hayhurst et al., 2018. |
| Pt.02 | 17 m | Severe Purkinje cell loss, Bergmann gliosis and GCL depletion; patchy astrogliosis in the dentate nucleus. |  |  |
| Pt.03 | 18 m | Almost total loss of Purkinje cells; severe Bergmann gliosis; GCL depletion; dentate nucleus neuron loss; loss of myelin. | Yes |  |
| Pt.04 | 2.8 y | Severe Purkinje cell loss and GCL atrophy. |  |  |
| Pt.05 | 3.2 y | Focal lesions of total Purkinje cell loss, severe thinning of MCL, atrophy of GCL and gliosis. | Yes |  |
| Pt.06 | 4 y | Mild Purkinje cell loss; marked GCL depletion; atrophic MCL. |  |  |
| Pt.07 | 7 y | Severe Purkinje cell loss, GCL depletion and Bergmann gliosis; dentate nucleus neuron loss. |  | Hayhurst et al., 2018. Jellinger et al., 1970. |
| Pt.08 | 11.9 | Mild Purkinje cell loss; mild Bergmann gliosis. |  |  |
| Pt.09 | 12.5 | Patchy Purkinje cell loss; severe Bergmann gliosis. |  | Hayhurst et al., 2018. |
| Pt.10 | 14 y | Patchy Purkinje cell loss; GCL depletion; severe Bergmann gliosis. |  | Hayhurst et al., 2018. |
| Pt.11 | 23 y | Moderate to severe patchy Purkinje cell loss with Bergmann gliosis. |  | Ng et al., 2022. |
| Pt.12 | 24 y | Focal lesion of total Purkinje cell loss, severe thinning of MCL, atrophy of GCL and gliosis; severe dentate nucleus neuron loss; focal demyelination. | Yes | Chrysostomou et al. 2016. Lax et al., 2012.A. Lax et al., 2012.B. Ng et al., 2022. |
| Pt.13 | 28 y | Focal lesions of total PK cell loss, severe thinning of MCL, atrophy of GCL and gliosis; severe vacuolisation of subcortical white matter. | Yes | Ng et al. 2022. |
| Pt.14 | 46 y | Patchy moderate loss of Purkinje cells; moderate loss of neurons in the dentate nucleus; mild diffuse loss of myelin staining. |  |  |
| Pt.15 | 50 y | Lesion characterised by severe atrophy of PCL, MCL and GCL; severe loss of Purkinje cells; demyelination of white matter; severe gliosis. | Yes | Ng. et al., 2022. |
| Pt.16 | 55 y | 5mm lesion characterised by microvacuolation, atrophy of MCL, Bergmann gliosis, total PK cell loss and GCL depletion; patchy widespread Purkinje cell loss; dentate neuron loss; mild myelin loss. | Yes | Chrysostomou et al. 2016. Ng et al., 2022. |
| Pt.17 | 59 y | Single infarct affecting the MCL, PCL, GCL; moderate PK cell loss; eosinophilic neurons; focal minor dentate neuron loss. | Yes  (Neuropathology  Report) | Chrysostomou et al. 2016. Lax et al., 2012.A. Ng et al., 2022. |
| Pt.18 | 79 y | Patchy Purkinje cell loss. |  | Chrysostomou et al. 2016. Ng et al., 2022. |
| Pt.19 | 20 y | Inferior-posterior cerebellum severely affected by large focal lesion characterised by subtotal Purkinje cell loss and atrophy of the MCL and GCL; white matter demyelination; dentate nucleus neuron loss. | Yes | Chrysostomou et al. 2016. Lax et al., 2012.A. Lax et al., 2012.B. Ng et al., 2022. |
| Pt.20 | 42 y | Focal Purkinje cell loss and microvacuolation of the PCL; GCL loss; diffuse demyelination; microgliosis affecting dentate nucleus. |  | Lax et al., 2012.A. Lax et al., 2012.B. Ng et al., 2022. |
| Pt.21 | 45 y | Atrophy of GCL and MCL, most severely affecting superior lobes; areas of focal Purkinje cell loss but density overall preserved; demyelination in dentate nucleus. |  | Chrysostomou et al. 2016. Lax et al., 2012.A. Lax et al., 2012.B. Ng et al., 2022. |
| Pt.22 | 64 y | Mild to moderate loss of Purkinje cells associated with Bergmann gliosis. |  | Ng et al., 2022. |
| Pt.23 | 31 y | Moderate Purkinje cell loss; severe loss of neurons in the dentate nucleus. |  |  |
| Pt.24 | 42 y | Severe multi-focal loss of Purkinje cells accompanied by focal microvacuolation; mild focal loss of GCL cells and thinning of MCL. |  | Chrysostomou et al. 2016. Lax et al., 2012.A. Lax et al., 2012.B. Ng et al., 2022. |
| Pt.25 | 58 y | Patchy Purkinje cell loss; focal thinning of MCL; moderate to severe loss of neurons in dentate nucleus. |  | Chrysostomou et al. 2016. Lax et al., 2012.B. Ng et al., 2022. |
| Pt.26 | 64 y | Moderate Purkinje cell loss; mild focal loss of neurons in the dentate nucleus. | Yes |  |
| Pt.27 | 34 y | 10mm lesion characterised by severe thinning of the MCL, almost complete loss of PK cells but intact GCL; dentate nucleus neuron loss. | Yes  (Neuropathology Report) | Lax et al., 2012.A. Lax et al., 2012.B. Ng et al., 2022. |
| Pt.28 | 55 y | Focal Purkinje cell loss and profound loss of dentate nucleus neurons; mild microvacuolation of the MCL. |  | Chrysostomou et al. 2016. Lax et al., 2012.A. Lax et al., 2012.B. Ng et al., 2022. |

**Abbreviations**: *PCL* Purkinje cell layer; *GCL* Granular cell layer; *MCL* Molecular cell layer. Neuropathological features of the cerebellum have been summarised from post-mortem neuropathology reports and assessment of histological stains using available FFPE cerebellar tissues.

**References for previous cerebellar publications (Supplementary Table 6)**:

Chrysostomou A, Grady JP, Laude A, Taylor RW, Turnbull DM, Lax NZ (2016) **Investigating complex I deficiency in Purkinje cells and synapses in patients with mitochondrial disease**. Neuropathology and applied neurobiology 42: 477-492 Doi 10.1111/nan.12282

Hayhurst H, Anagnostou, M. E., Bogle, H. J., Grady, J. P., Taylor, R. W., Bindoff, L. A., McFarland, R., Turnbull, D. M. & Lax, N. Z. (2018) **Dissecting The Neuronal Vulnerability Underpinning Alpers' Syndrome: A Clinical And Neuropathological Study**. Brain Pathology 29: 97-113.

Jellinger K, Seitelberger F (1970) **Spongy glio-neuronal dystrophy in infancy and childhood**. Acta Neuropathologica 16: 125-140 Doi 10.1007/BF00687667

Lax NZ, Hepplewhite PD, Reeve AK, Nesbitt V, McFarland R, Jaros E, Taylor RW, Turnbull DM (2012.A) **Cerebellar ataxia in patients with mitochondrial DNA disease: a molecular clinicopathological study**. J Neuropathol Exp Neurol 71: 148-161 Doi 10.1097/NEN.0b013e318244477d

Lax NZ, Pienaar IS, Reeve AK, Hepplewhite PD, Jaros E, Taylor RW, Kalaria RN, Turnbull DM (2012.B) **Microangiopathy in the cerebellum of patients with mitochondrial DNA disease**. Brain 135: 1736-1750 Doi 10.1093/brain/aws110

Ng Y, Lax NZ, Blain A, Erskine D, Baker M, Polvikoski T, Thomas R, Morris C, Lai M, Whittaker Ret al (2022) **Forecasting stroke-like episodes and outcomes in mitochondrial disease**. Brain 145: 542-554


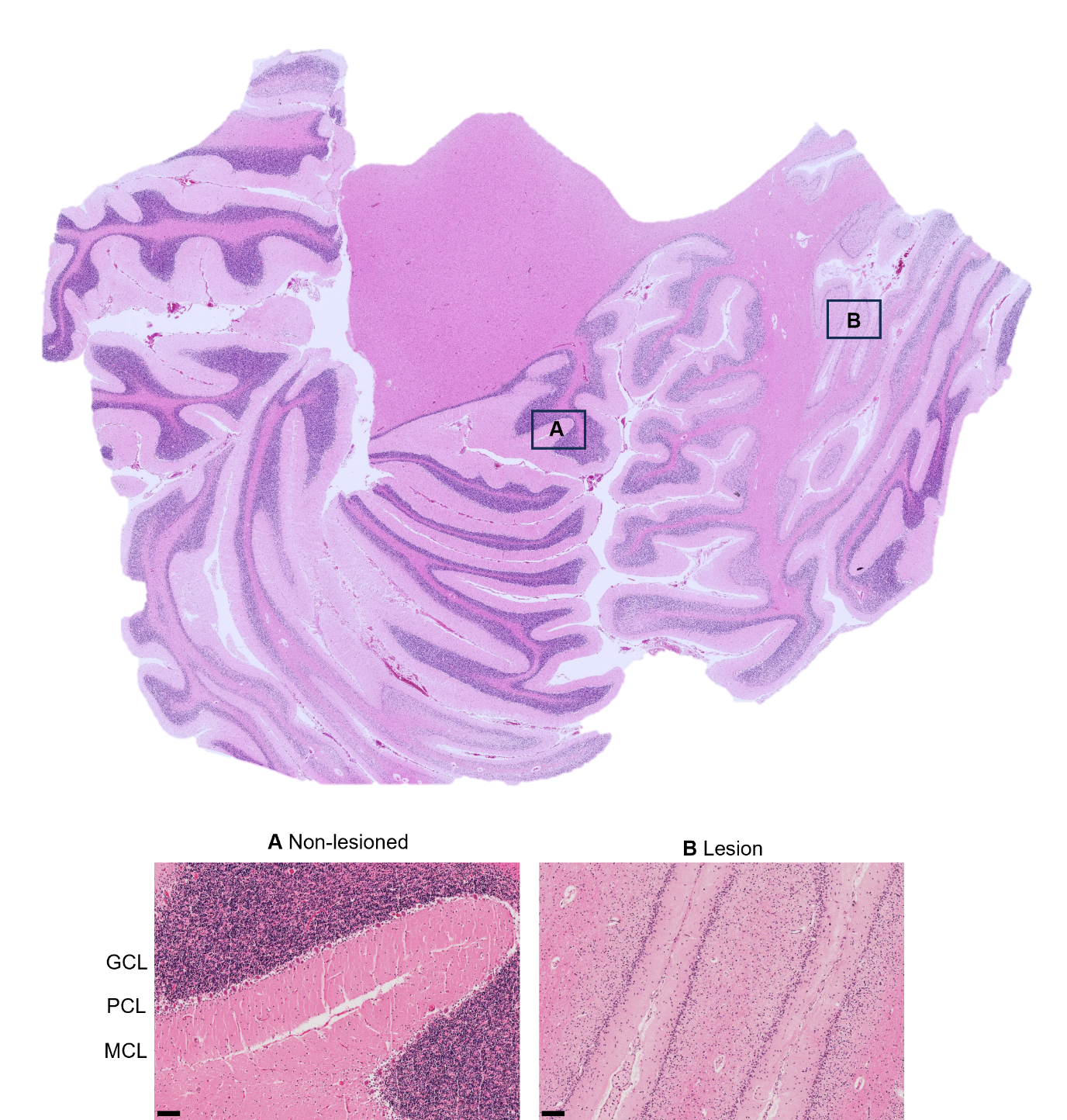


**Supplementary Fig. 1 Focal necrotic lesion in the cerebellar cortex of a patient with Alpers’ syndrome (Pt.5).** Haematoxylin and Eosin (H&E) histological stain demonstrates preserved cerebellar cortex (**a**), with intact Purkinje cell layer (PCL), granular cell layer (GCL) and molecular cell layer (MCL), in comparison to lesioned cerebellar cortex (**b**) which is affected by cortical thinning, absence of Purkinje cells, Bergmann gliosis and decreased cell density within the GCL. Scale bars = 100 μm


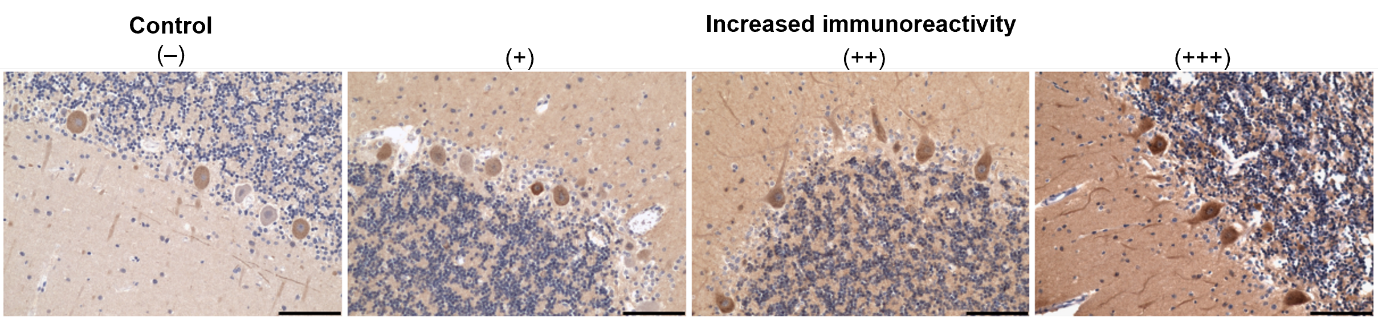


**Supplementary Fig. 2 Qualitative scoring system used to assess the immunoreactivity of LC3B protein expression in the cerebellar cortex.** Representative staining of LC3B (autophagosome membrane protein) in control cerebellar Purkinje cells is presented (**–**) in comparison to LC3B staining in cerebellar tissues from patients with primary mitochondrial disease (**+**, **++**, **+++**). The density and intensity of LC3B-immunoreactive Purkinje cells is increased from (**–**) to (**+++**). A similar scoring system was used to assess other markers (BNIP3, p62 and c-Fos) in cerebellar tissues. Scale bars = 100 μm


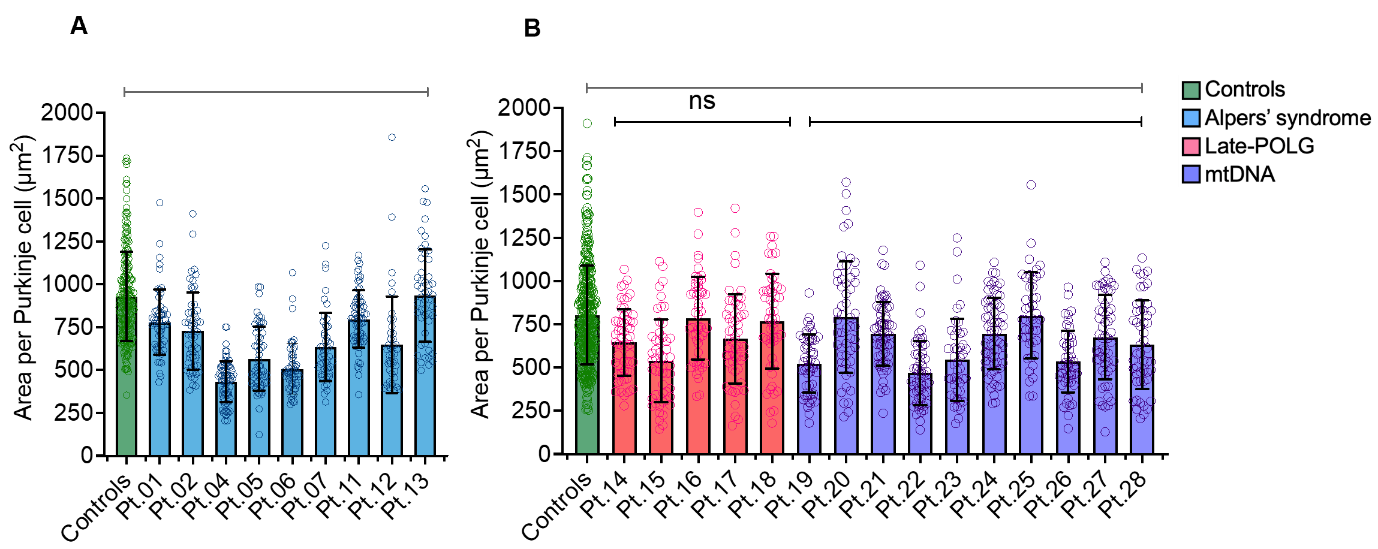


**Supplementary Fig. 3 Atrophy of Purkinje cells in mitochondrial disease.** The area (μm^2^) of individual Purkinje cells from patients with Alpers’ syndrome (**a,** blue) and late-onset POLG-related disease (pink) and mtDNA disease (purple) (**b**), relative to age-matched control cases (green) are presented. Purkinje cells were imaged at x20 magnification and were identified based on morphology, localisation within the linear Purkinje cell layer, and positive parvalbumin and porin staining. Analysis of data using a linear mixed regression model revealed at the group level, Purkinje cells are significantly smaller in patients with Alpers’ syndrome and late-onset mitochondrial disease relative to control Purkinje cells. ***** *P* < 0.05, ****** *P* < 0.01

**
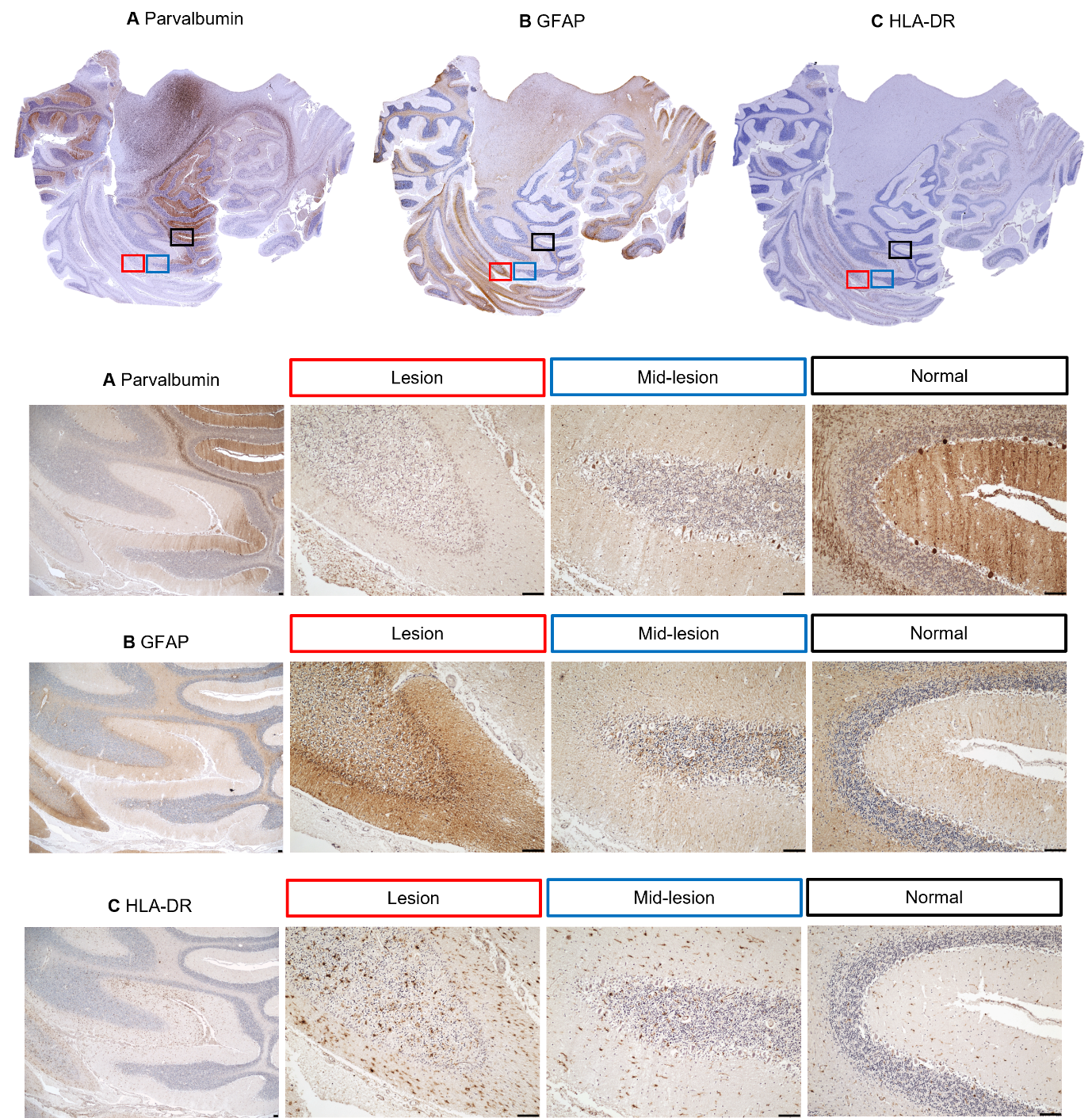
**

**Supplementary Fig. 4 Lesioned cerebellar cortex from a patient with Alpers’ syndrome.** Focal lesioned cerebellar cortex (red box) from Patient 5 (p.[Cys418Arg]/p.[Ala467Thr]) is characterised by a loss of parvalbumin+ immunoreactivity (**a**) and increased GFAP+ (**b**) and HLA-DR+ (**c**) immunoreactivity. Adjacent cortex (blue box, mid-lesion) demonstrates an intermediate level of immunoreactivity of parvalbumin, GFAP and HLA-DR compared to non-lesioned normal cortex (black box) and the lesioned cortex. Scale bars = 100 μm


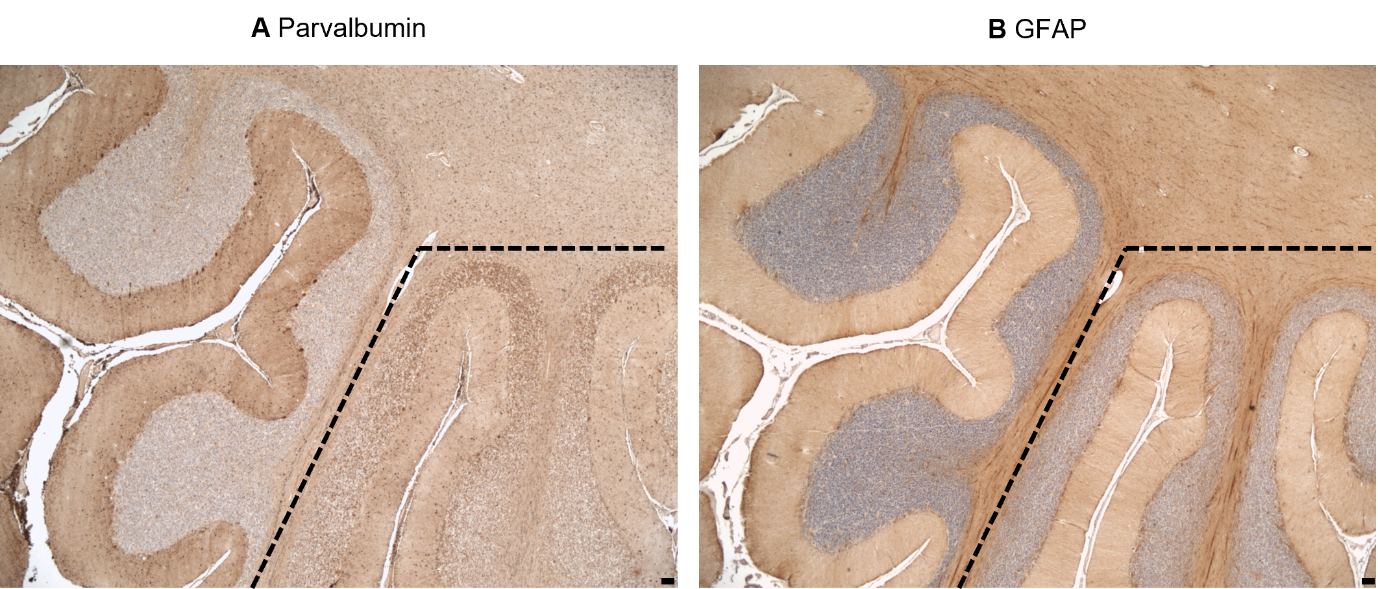


**Supplementary Fig. 5 Lesioned cerebellar cortex from an adult patient harbouring the m.8344 A>G pathogenic variant.** Representative images of a focal lesion (black dashed outline) from an adult patient with mtDNA disease (Patient 26) demonstrates a focal loss of parvalbumin+ immunoreactivity (**a**), however, within this region, GFAP+ immunoreactivity appears to be similar to adjacent non-lesioned cortex (**b**). Scale bars = 100 μm

**
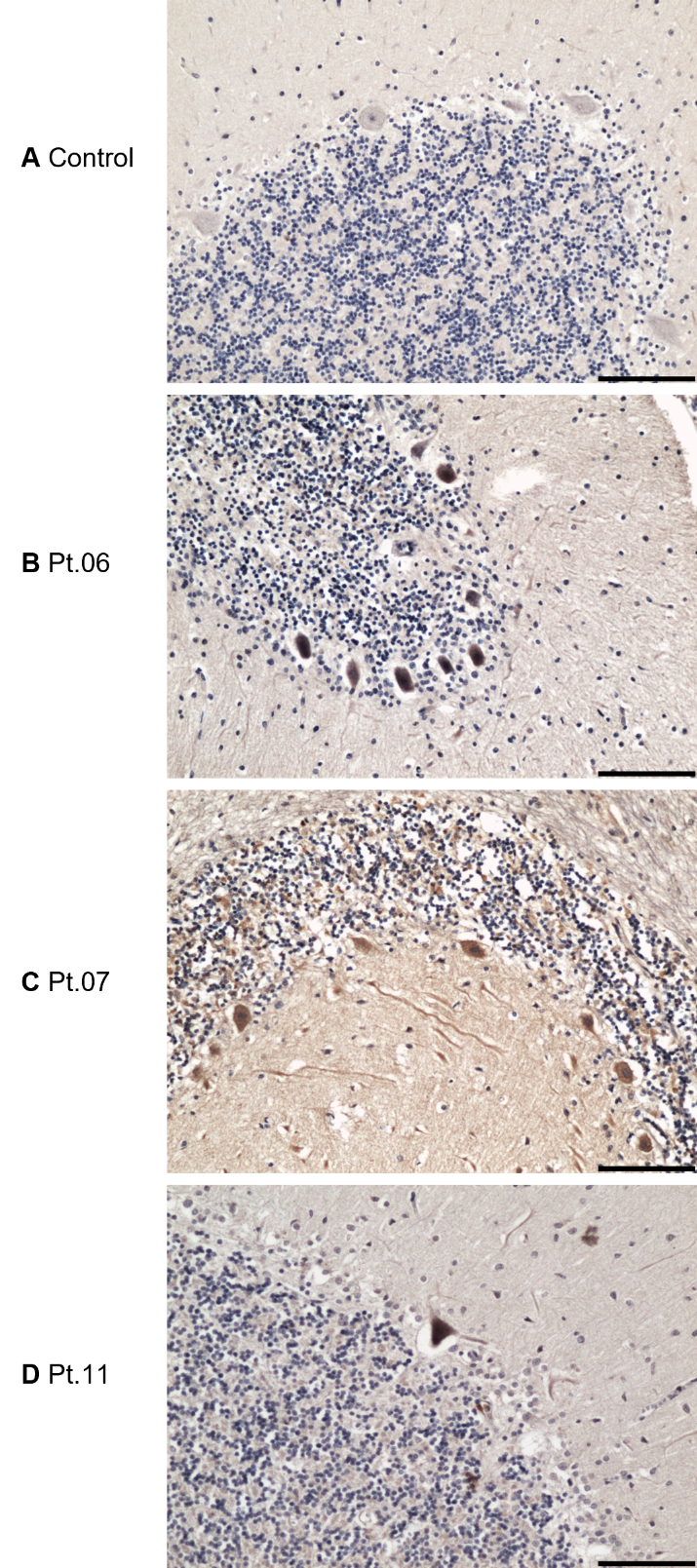
**

**Supplementary Fig. 6 c-Fos immunoreactivity in the cerebellar cortex.** (**a**) Representative control tissue demonstrates minimal c-Fos immunoreactivity. (**b**) Cerebellar tissues from patients with early-onset primary mitochondrial disease (Pt.06, Pt.07, Pt.11) demonstrate increased c-Fos immunoreactivity, predominantly localised within Purkinje cells. Scale bars = 100 μm


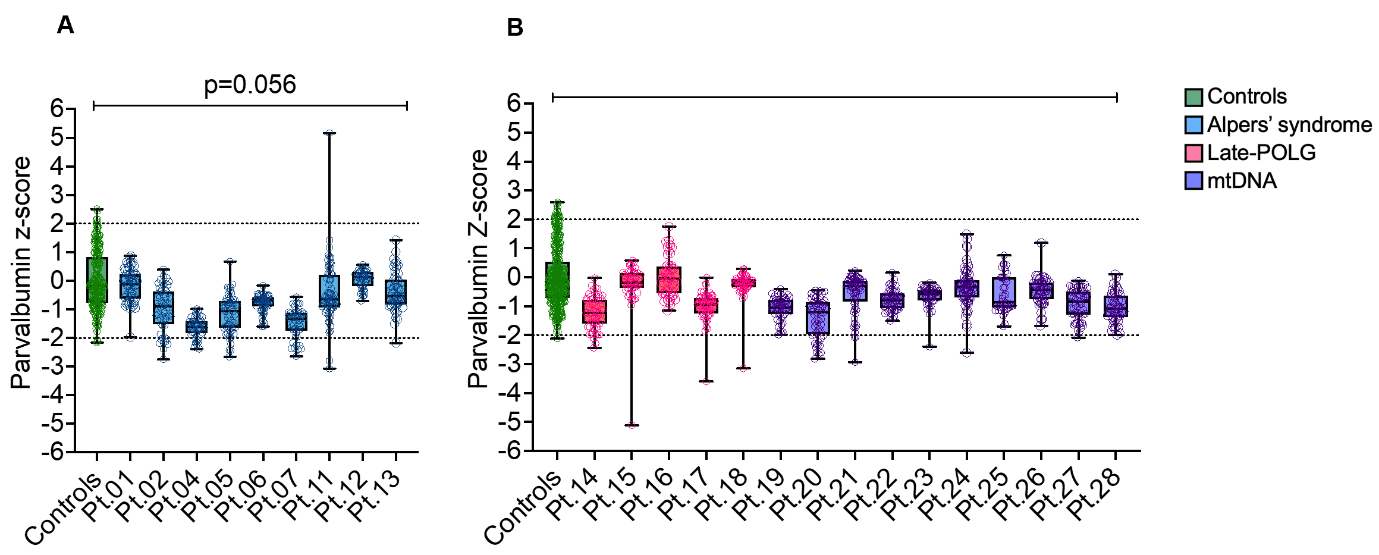


**Supplementary Fig. 7 Changes in parvalbumin expression in Purkinje neurons in patients with mitochondrial disease.** (**a**) PV z-scores in Purkinje neurons per case for patients with Alpers’ syndrome (blue). PV expression was non-significantly decreased in Alpers’ syndrome group in comparison to controls (*P* = 0.0564, linear mixed-effects model). (**b**) PV z-scores in Purkinje neurons per case for patients with adult-onset POLG (pink) and mtDNA disease (purple) indicate an overall decrease (*P* = 0.0139, linear mixed-effects model). PV expression is significantly decreased in mtDNA disease group and not late-onset POLG group (*P* = 0.0330 and 0.3033, respectively, linear mixed-effects model)


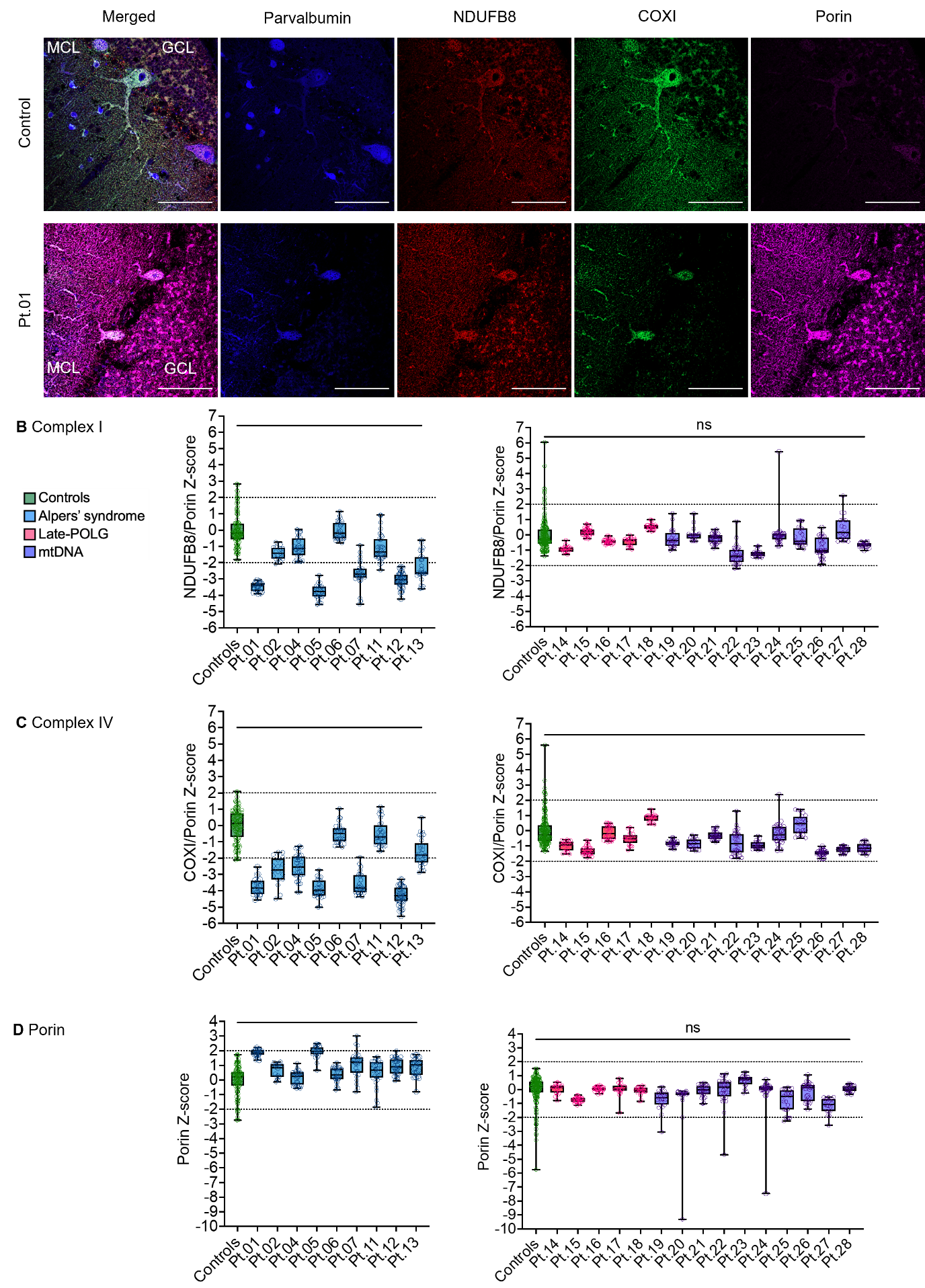


**Supplementary Fig. 8 Mitochondrial oxidative phosphorylation protein deficiencies in the granular cell layer in mitochondrial disease.** (**a**) Representative confocal images demonstrating quadruple immunofluorescence for parvalbumin (blue; Purkinje cell marker), NDUFB8 (red; complex I subunit), COXI (green; complex IV subunit) and porin (purple; mitochondrial mass marker) within the cerebellar cortex. (**b**) NDUFB8 z-scores in granule cell layer per case for patients with Alpers’ syndrome (blue), adult-onset POLG (pink) and mtDNA disease (purple). Complex I deficiencies were significant in Alpers’ syndrome, but not in adult-onset mitochondrial disease (*P* = 0.0119 and 0.2109, respectively, linear mixed-effects model). (**c**) COXI z-scores in Purkinje neurons were significantly decreased in Alpers’ syndrome and in adult-onset mitochondrial disease (*P* = 0.0056 and 0.0427, respectively, linear mixed-effects model). (**d**) Porin z-scores were statistically greater in Alpers’ syndrome and remained unaltered in adult mitochondrial disease (*P* = 0.0344 and 0.2532, respectively, linear mixed-effects model)


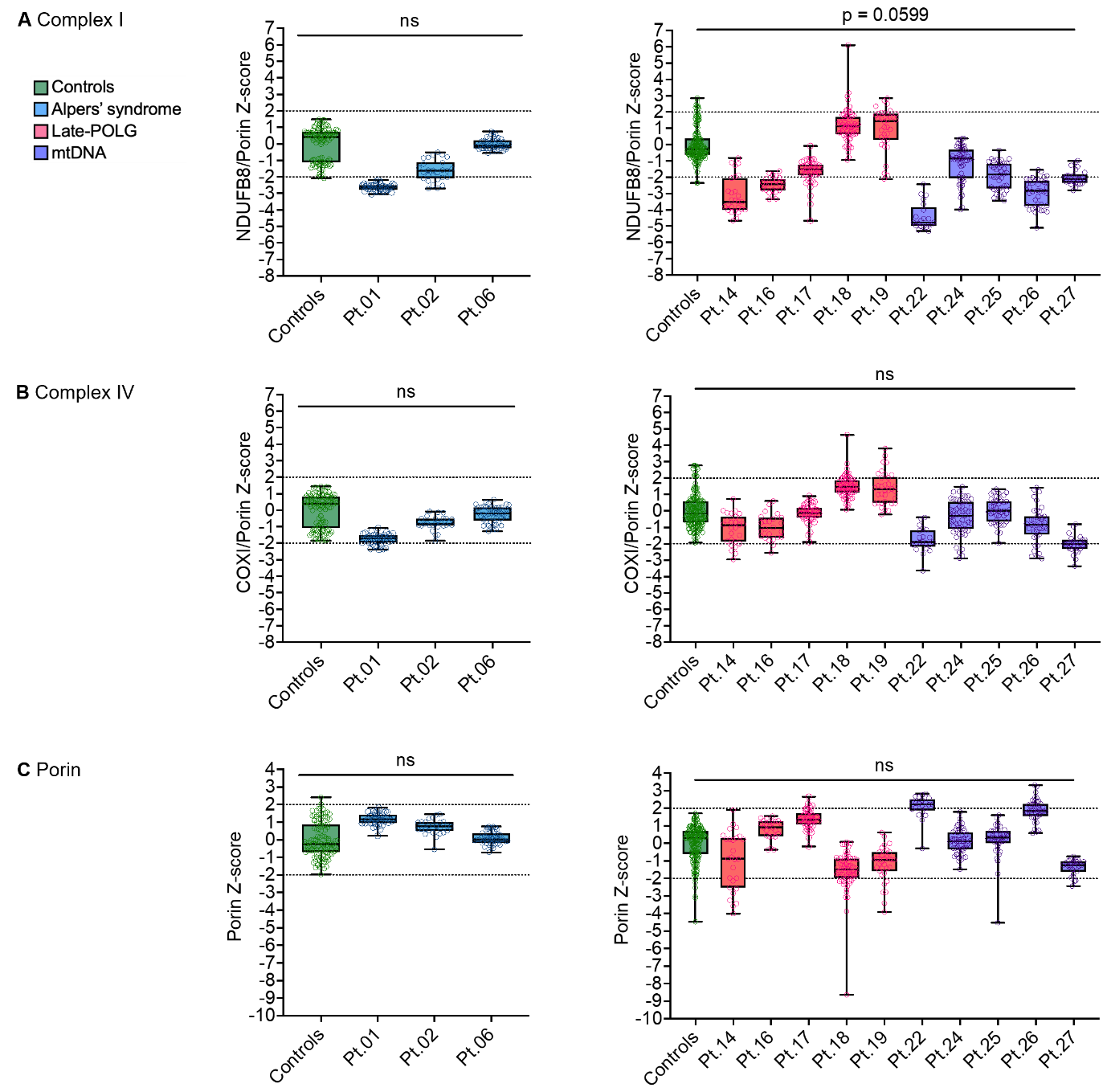


**Supplementary Fig. 9 Mitochondrial oxidative phosphorylation protein changes in the dentate nucleus in mitochondrial disease**. Dentate nucleus neurons did not demonstrate statistically significant changes in (**a**) NDUFB8, (**b**) COXI or (**c**) porin protein expression in either the Alpers’ syndrome (*P* = 0.23, 0.291 and 0.384, respectively, linear mixed-effects model) or late-onset mitochondrial disease patient cohorts (*P* = 0.0599, 0.3843 and 0.8122, respectively, linear mixed-effects model).


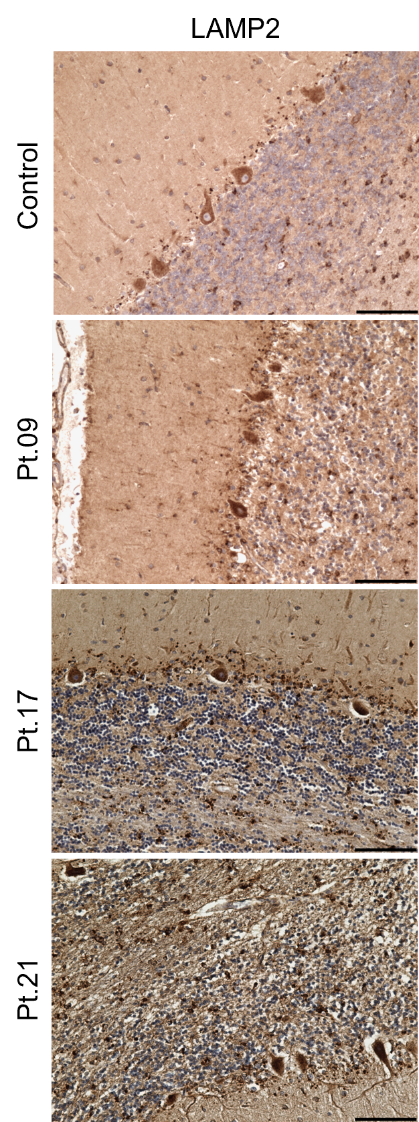


**Supplementary Fig. 10 LAMP2 immunoreactivity in the cerebellum**. Representative images of the lysosomal marker LAMP2 (lysosome-associated membrane protein 2) demonstrate similar immunoreactivity in control and primary mitochondrial disease patient Purkinje cells, suggesting that LAMP2 protein expression is unaltered. Scale bars = 100 μm

**Supplementary Table 7**: Analysis of OXPHOS immunofluorescence data using a linear mixed effects model.

| **Group comparisons** | | | **P value** | **Mean value** | | | |
| --- | --- | --- | --- | --- | --- | --- | --- |
|  |  |  |  | **Control group** | **Alpers** | **Late-POLG** | **mtDNA** |
| **Purkinje cell OXPHOS data:** | | | | | | | |
| **Young cohort comparisons** | NDUFB8 z-score | Alpers vs Young Controls | **0.0179** | 0.0300 | -2.03 |  |  |
|  | COXI z-score |  | **0.0230** | 0.0232 | -2.3876 |  |  |
|  | Porin z-score |  | **0.0122** | -0.0187 | 1.5672 |  |  |
|  | PV z-score |  | **0.0564** | -0.0105 | -0.8581 |  |  |
|  | Area |  | **0.0125** | 929 | 669 |  |  |
| **Older cohort comparisons** | NDUFB8 z-score | Late-POLG + mtDNA vs Older Controls | **0.0281** | 0.0238 |  | -0.885 | |
|  | COXI z-score |  | **0.0918** | 0.0426 |  | -0.751 | |
|  | Porin z-score |  | **0.474** | -0.0115 |  | -0.266 | |
|  | PV z-score |  | **0.0139** | -0.0306 |  | -0.700 | |
|  | Area |  | **0.0086** | 807 |  | 652 | |
|  | NDUFB8 z-score | Control vs mtDNA | **0.0821** | 0.0239 |  | -0.750 | -0.953 |
|  |  | Control vs Late-POLG | **0.319** |  |  |  |  |
|  |  | mtDNA vs Late-POLG | **0.917** |  |  |  |  |
|  | COXI z-score | Control vs mtDNA | **0.338** | 0.0426 |  | -0.895 | -0.679 |
|  |  | Control vs Late-POLG | **0.292** |  |  |  |  |
|  |  | mtDNA vs Late-POLG | **0.931** |  |  |  |  |
|  | Porin z-score | Control vs mtDNA | **0.743** | -0.0115 |  | -0.200 | -0.300 |
|  |  | Control vs Late-POLG | **0.916** |  |  |  |  |
|  |  | mtDNA vs Late-POLG | **0.975** |  |  |  |  |
|  | PV z-score | Control vs mtDNA | **0.0330** | -0.0306 |  | -0.538 | -0.781 |
|  |  | Control vs Late-POLG | **0.303** |  |  |  |  |
|  |  | mtDNA vs Late-POLG | **0.745** |  |  |  |  |
|  | Area | Control vs mtDNA | **0.0245** | 807 |  | 681 | 637 |
|  |  | Control vs Late-POLG | **0.211** |  |  |  |  |
|  |  | mtDNA vs Late-POLG | **0.810** |  |  |  |  |
| **PMD group comparisons** | NDUFB8 z-score | Alpers vs mtDNA | **0.111** |  | -2.03 | -0.750 | -0.953 |
|  |  | Alpers vs Late-POLG | **0.120** |  |  |  |  |
|  |  | mtDNA vs Late-POLG | **0.940** |  |  |  |  |
|  | COXI z-score | Alpers vs mtDNA | **0.0406** |  | -2.388 | -0.895 | -0.679 |
|  |  | Alpers vs Late-POLG | **0.167** |  |  |  |  |
|  |  | mtDNA vs Late-POLG | **0.958** |  |  |  |  |
|  | Porin z-score | Alpers vs mtDNA | **0.0005** |  | 1.567 | -0.2 | -0.299 |
|  |  | Alpers vs Late-POLG | **0.0049** |  |  |  |  |
|  |  | mtDNA vs Late-POLG | **0.977** |  |  |  |  |
|  | PV z-score | Alpers vs mtDNA | **0.941** |  | -0.858 | -0.538 | -0.781 |
|  |  | Alpers vs Late-POLG | **0.506** |  |  |  |  |
|  |  | mtDNA vs Late-POLG | **0.663** |  |  |  |  |
|  | Area | Alpers vs mtDNA | **0.868** |  | 669 | 681 | 637 |
|  |  | Alpers vs Late-POLG | **0.983** |  |  |  |  |
|  |  | mtDNA vs Late-POLG | **0.818** |  |  |  |  |
| **Granular cell layer OXPHOS data:** | | | | | | | |
| **Young cohort comparisons** | NDUFB8 z-score | Alpers vs Young Controls | **0.0119** | 0.0699 | -2.100 |  |  |
|  | COXI z-score |  | **0.0056** | 0.0650 | -2.610 |  |  |
|  | Porin z-score |  | **0.0344** | -0.0614 | 0.924 |  |  |
| **Older cohort comparisons** | NDUFB8 z-score | Late-POLG + mtDNA vs Older Controls | **0.2109** | 0.0302 |  | -0.343 | |
|  | COXI z-score |  | **0.0427** | 0.0524 |  | -0.624 | |
|  | Porin z-score |  | **0.253** | 0.00552 |  | -0.274 | |
|  | NDUFB8 z-score | Control vs mtDNA | **0.360** | 0.0303 |  | -0.205 | -0.412 |
|  |  | Control vs Late-POLG | **0.819** |  |  |  |  |
|  |  | mtDNA vs Late-POLG | **0.852** |  |  |  |  |
|  | COXI z-score | Control vs mtDNA | **0.087** | 0.0524 |  | -0.424 | -0.725 |
|  |  | Control vs Late-POLG | **0.505** |  |  |  |  |
|  |  | mtDNA vs Late-POLG | **0.750** |  |  |  |  |
|  | Porin z-score | Control vs mtDNA | **0.400** | 0.00545 |  | -0.139 | -0.342 |
|  |  | Control vs Late-POLG | **0.894** |  |  |  |  |
|  |  | mtDNA vs Late-POLG | **0.795** |  |  |  |  |
| **PMD group comparisons** | NDUFB8 z-score | Alpers vs mtDNA | **0.0016** |  | -2.100 | -0.205 | -0.412 |
|  |  | Alpers vs Late-POLG | **0.0032** |  |  |  |  |
|  |  | mtDNA vs Late-POLG | **0.909** |  |  |  |  |
|  | COXI z-score | Alpers vs mtDNA | **0.002** |  | -2.610 | -0.424 | -0.725 |
|  |  | Alpers vs Late-POLG | **0.003** |  |  |  |  |
|  |  | mtDNA vs Late-POLG | **0.857** |  |  |  |  |
|  | Porin z-score | Alpers vs mtDNA | **0.0001** |  | 0.924 | -0.139 | -0.342 |
|  |  | Alpers vs Late-POLG | **0.0057** |  |  |  |  |
|  |  | mtDNA vs Late-POLG | **0.776** |  |  |  |  |
| **Dentate nucleus neurons OXPHOS data:** | | | | | | | |
| **Young cohort comparisons** | NDUFB8 z-score | Alpers vs Young Controls | **0.230** | -0.0121 | -1.429 |  |  |
|  | COXI z-score |  | **0.291** | 0.291 | -1.215 |  |  |
|  | Porin z-score |  | **0.384** | 0.00195 | 0.664 |  |  |
|  | Area |  | **0.0304** | 681 | 352 |  |  |
| **Older cohort comparisons** | NDUFB8 z-score | Late-POLG + mtDNA vs Older Controls | **0.0599** | -0.0209 |  | -1.756 | |
|  | COXI z-score |  | **0.384** | 0.0897 |  | -0.430 | |
|  | Porin z-score |  | **0.812** | -0.0256 |  | 0.136 | |
|  | Area |  | **0.0170** | 602 |  | 460 | |
|  | NDUFB8 z-score | Control vs mtDNA | **0.165** | -0.0209 |  | -1.530 | -1.908 |
|  |  | Control vs Late-POLG | **0.365** |  |  |  |  |
|  |  | mtDNA vs Late-POLG | **0.928** |  |  |  |  |
|  | COXI z-score | Control vs mtDNA | **0.560** | 0.0897 |  | -0.179 | -0.598 |
|  |  | Control vs Late-POLG | **0.928** |  |  |  |  |
|  |  | mtDNA vs Late-POLG | **0.822** |  |  |  |  |
|  | Porin z-score | Control vs mtDNA | **0.909** | -0.0256 |  | -0.0962 | 0.291 |
|  |  | Control vs Late-POLG | **0.996** |  |  |  |  |
|  |  | mtDNA vs Late-POLG | **0.883** |  |  |  |  |
|  | Area | Control vs mtDNA | **0.0075** | 601 |  | 530 | 414 |
|  |  | Control vs Late-POLG | **0.434** |  |  |  |  |
|  |  | mtDNA vs Late-POLG | **0.117** |  |  |  |  |
| **PMD group comparisons** | NDUFB8 z-score | Alpers vs mtDNA | **0.921** |  | -1.429 | -1.530 | -1.908 |
|  |  | Alpers vs Late-POLG | **0.997** |  |  |  |  |
|  |  | mtDNA vs Late-POLG | **0.940** |  |  |  |  |
|  | COXI z-score | Alpers vs mtDNA | **0.915** |  | -1.215 | -0.179 | -0.598 |
|  |  | Alpers vs Late-POLG | **0.684** |  |  |  |  |
|  |  | mtDNA vs Late-POLG | **0.844** |  |  |  |  |
|  | Porin z-score | Alpers vs mtDNA | **0.916** |  | 0.664 | -0.0962 | 0.291 |
|  |  | Alpers vs Late-POLG | **0.736** |  |  |  |  |
|  |  | mtDNA vs Late-POLG | **0.893** |  |  |  |  |
|  | Area | Alpers vs mtDNA | **0.573** |  | 352 | 530 | 414 |
|  |  | Alpers vs Late-POLG | **0.0467** |  |  |  |  |

Analysis of OXPHOS immunofluorescence data using a linear mixed effects model. The intensity of NDUFB8, COXI and Porin within individual Purkinje cells, cells within the granular cell layer and individual dentate nucleus neurons were measured and compared between primary mitochondrial disease (PMD) patient groups and controls. The intensity of parvalbumin (PV) and area of Purkinje cells, and the area of individual dentate nucleus neurons were also measured and compared between groups.
The Alpers’ syndrome patients were compared to age-matched controls (‘Young Controls’), and the Late-POLG and mtDNA patients were grouped together and compared to age-matched controls (‘Older Controls’). The three PMD groups were also compared. Significant P values (*P* < 0.05) are highlighted in yellow.
